# A noncanonical parasubthalamic nucleus–to–extended amygdala circuit converts chronic social stress into anxiety

**DOI:** 10.1172/JCI188246

**Published:** 2025-08-15

**Authors:** Na Liu, Jun Wang, Huan Wang, Bin Gao, Zheng Lin, Tian-Le Xu, Shumin Duan, Han Xu

**Affiliations:** 1 Department of Psychiatry of The Second Affiliated Hospital and School of Brain Science and Brain Medicine, Zhejiang University School of Medicine, Hangzhou, China.; 2 Nanhu Brain-Computer Interface Institute, Hangzhou, China.; 3 Liangzhu Laboratory, MOE Frontier Science Center for Brain Science and Brain-Machine Integration, State Key Laboratory of Brain-Machine Intelligence, and; 4 NHC and CAMS Key Laboratory of Medical Neurobiology, Zhejiang University, Hangzhou, China.; 5 Collaborative Innovation Center for Brain Science, Department of Anatomy and Physiology, Shanghai Jiao Tong University School of Medicine, Shanghai, China.; 6 Lingang Laboratory, Shanghai, China.

**Keywords:** Cell biology, Neuroscience, Behavior, Ion channels, Neurological disorders

## Abstract

Anxiety disorders pose a substantial threat to global mental health, with chronic stress identified as a major etiologic factor. Over the past few decades, extensive studies have revealed that chronic stress induces anxiety states through a distributed neuronal network of interconnected brain structures. However, the precise circuit mechanisms underlying the transition from chronic stress to anxiety remain incompletely understood. Employing the chronic social defeat stress (CSDS) paradigm in mice, we uncovered a critical role of the parasubthalamic nucleus (PSTh) in both the induction and expression of anxiety-like behavior. The anxiogenic effect was mediated by an excitatory trisynaptic circuitry involving the lateral parabrachial nucleus (LPB), PSTh, and bed nucleus of the stria terminalis (BNST). Furthermore, CSDS downregulated Kv4.3 channels in glutamatergic neurons of the PSTh. Reexpression of these channels dampened neuronal overexcitability and alleviated anxiety-like behavior in stressed animals. In parallel with the well-known anxiety network centered on the amygdala, here we identify a noncanonical LPB-PSTh-BNST pathway in the transformation of stress into anxiety. These findings suggest that the PSTh may serve as a potential therapeutic target for anxiety-related disorders.

## Abstract 2

Chronic social stress induces hyperexcitability of PSTh glutamatergic neurons by downregulating Kv4.3 channels, leading to anxiety via a non-canonical PSTh to extra-amygdala circuitry

## Graphical abstract

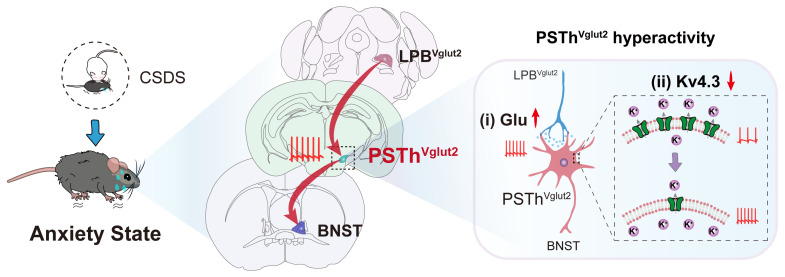

## Introduction

Anxiety disorders represent the most prevalent class of psychiatric disorders, with a lifetime morbidity rate exceeding 28% (1–3). These disorders have become an intractable mental health issue due to their severe impact on individuals’ emotional, physical, and behavioral well-being, often resulting in substantial disability (4, 5). However, therapeutic options for anxiety disorders remain inadequate, partly due to a limited understanding of their neural substrates (6–8).

Anxiety is characterized as a negative emotional state marked by intense worry, heightened arousal, and increased vigilance (9–11). Growing evidence supports the notion that anxiety is regulated by multiple distributed brain regions and their interconnected neural circuits. Key areas include the amygdala (12, 13), medial prefrontal cortex (mPFC) (14, 15), and ventral hippocampus (16–18), all of which play central roles in modulating anxious state. Notably, hyperactivity of the amygdala, partly due to impaired inhibitory influence from the mPFC and ventral hippocampus, is believed to contribute to the pathophysiology of anxiety disorders (19–22). Recent evidence indicates that disturbances in an extra-amygdala region, i.e., the bed nucleus of the stria terminalis (BNST), as an efferent target of the hyperactive amygdala, contribute to the manifestation of anxiety disorders (23–25). Additionally, structures such as the paraventricular nucleus of the hypothalamus (PVN), medial preoptic area (mPOA), and lateral septum have also been implicated in generation of anxiety conditions (11, 26, 27). Despite significant progress made in recent decades, a comprehensive understanding of the complex anxiety network remains elusive. In particular, the roles of brain structures outside the limbic system centered on the amygdala in the induction and maintenance of anxiety are still underexplored.

The parasubthalamic nucleus (PSTh) is a small excitatory nucleus located in the posterior part of the lateral hypothalamus (28), and its physiological functions are incompletely understood. Recently, studies have begun to reveal its crucial roles in feeding behavior, thermoregulation, exploration-associated wakefulness, and innate fear (29–32). Intriguingly, the PSTh exhibits strong anatomical connectivity with key anxiety-regulating structures, including the BNST, central amygdala (CeA), and so on (28). However, whether the PSTh contributes to the induction and expression of anxiety-like behaviors and the specific circuit mechanisms underlying its potential role in anxiety regulation remain unclear.

Chronic stress represents one of the most common contributors to anxiety disorders (2, 33, 34). In the present study, we demonstrate that the PSTh is readily activated by acute stressors and is essential for transforming external chronic stress into an internal anxiety state in mice. The PSTh receives glutamatergic inputs from the lateral parabrachial nucleus (LPB) and sends direct excitatory projections onto the BNST. The LPB-PSTh-BNST excitatory trisynaptic circuitry plays a crucial role in anxiety-like behavior induced by chronic social defeat stress (CSDS). Furthermore, chronic stress increased the excitability of PSTh glutamatergic neurons by downregulating Kv4.3 expression, which subsequently induced anxiety. Conversely, restoring Kv4.3 expression in PSTh glutamatergic neurons normalized their overexcitability and produced an anxiolytic effect in CSDS mice. These findings reveal a significant excitatory trisynaptic circuitry involving the PSTh in the transformation of chronic social stress into anxiety. Additionally, our studies suggest that the PSTh could serve as a promising therapeutic target for the prevention and treatment of anxiety-related disorders.

## Results

### The PSTh is robustly activated by various acute stressors.

**Figure 1:**
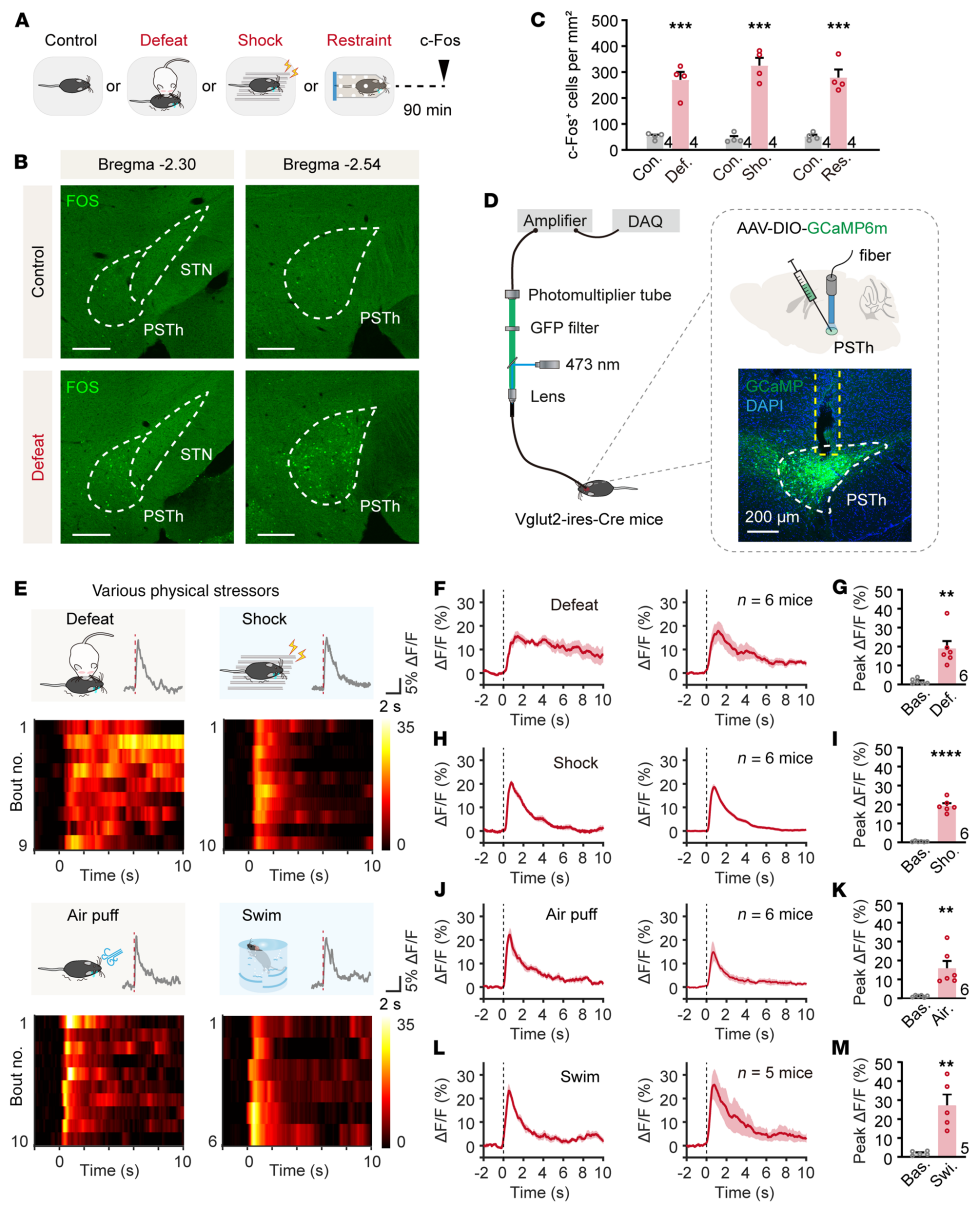
PSTh glutamatergic neurons are activated by various acute stressors. (**A**) Schematic diagram of c-Fos staining. (**B**) c-Fos expression in the PSTh from a control (top) and a social defeat–exposed mouse (bottom). Scale bars: 200 μm. (**C**) The number of c-Fos-positive cells in control (*n* = 4) and stressed mice (*n* = 4). (**D**) Schematic illustration of fiber photometry recordings and representative image of GCaMP6m expression in PSTh glutamatergic neurons. Scale bar: 200 μm. (**E**) Representative raw traces and heatmaps showing GCaMP6m fluorescence changes of PSTh^Vglut2^ neurons evoked by various stressors. The red line indicates stimulus onset. (**F**) The peri-event plot shows average calcium transients in a social defeat–exposed mouse (left) or the entire group (right, *n* = 6). The thick line indicates the mean, and the shaded area indicates SEM. The dotted line marks onset of social defeat. (**G**) Statistical comparison of peak fluorescence signals before and after social defeat (*n* = 6). (**H**–**M**) The same as **F** and **G** but for Ca^2+^ responses to electrical shock (**H** and **I**; *n* = 6), air puff (**J** and **K**; *n* = 6), or forced swim (**L** and **M**; *n* = 5). Data are shown as the mean ± SEM. **P < 0.01; ***P < 0.001; ****P < 0.0001; 2-tailed unpaired *t* test.

Given that stress is a key etiologic factor in anxiety induction, brain structures responsible for stress-induced anxiety are likely responsive to acute negative stimuli (11). Therefore, we first subjected male mice to 10 minutes of social defeat stress, a commonly used stressor known to induce a high level of anxiety (35–37) (Figure 1A). We then performed whole-brain staining for c-Fos protein, an immediate-early gene product, to identify potential brain regions involved in anxiety generation. We observed a significant increase in the number of c-Fos–positive neurons in several areas known to contribute to anxiety-like behavior, including the mPFC, BNST, paraventricular thalamic nucleus, basolateral amygdala, CeA, PVN, periaqueductal gray (PAG), and LPB (10) (Supplemental Figure 1; supplemental material available online with this article; https://doi.org/10.1172/JCI188246DS1). Intriguingly, in addition to those known anxiety-related brain structures, we found a significant increase in c-Fos–positive neurons in the PSTh of subthalamic areas of stressed animals compared with controls (Figure 1, B and C). Moreover, this observation was consistent across other acute stressors, including electric foot shocks (0.6 mA, 0.5 s) and physical restraint (2 hours) (Figure 1C). These data indicate that PSTh neurons are responsive to various anxiogenic stressors.

To assess the real-time activity of PSTh neurons in response to anxiogenic stressors, we directly measured their activity using fiber photometry in freely moving mice. Since the PSTh predominantly consists of glutamatergic neurons expressing vesicular glutamate transporter 2 (Vglut2) (28), we stereotaxically infused Cre-inducible AAV-DIO-GCaMP6m into the PSTh of Vglut2-Cre mice and implanted an optical fiber directly above the PSTh (Figure 1D). We then recorded fluorescence signals of PSTh glutamatergic neurons in mice subjected to various acute stressors. Notably, we observed a robust increase in fluorescence signals (as represented in ΔF/F) each time the experimental mouse was attacked by an aggressive CD1 mouse (Figure 1, E–G). Similarly, such an increase in fluorescence signals was also observed when the experimental mouse received an electric foot shock or an air puff or was forced to swim (Figure 1, E and H–M). These data reveal that PSTh glutamatergic neurons are instantaneously activated by various stressors, indicating their role in encoding aversive stimuli.

### The PSTh is required for anxiety induction by chronic stress.

Chronic social stress is a significant risk factor for anxiety disorder in humans (33, 35, 38). To induce anxiety-like behaviors in mice, we employed the CSDS paradigm (36). In this approach, individual mice were defeated by a new aggressive CD1 mouse (~3 attacks per defeat session) each day. Following each day of defeat, the defeated mouse was cohoused with the aggressor, separated by a clear perforated divider, subjecting it to continuous psychological stress. After 7 consecutive days of defeat, we measured the animals’ anxiety-like behaviors with 2 standard behavioral assays: the elevated plus maze (EPM) test and open field test (OFT) (39) (Supplemental Figure 2, A and B). Consistent with previous reports (33, 35), defeated mice spent significantly less time in open arms of the EPM (Supplemental Figure 2, C–G) and the center zone of the OFT (Supplemental Figure 2, H–L). Thus, CSDS effectively induced robust anxiety-like behaviors in mice, ensuring it was a suitable paradigm for the present study.

**Figure 2:**
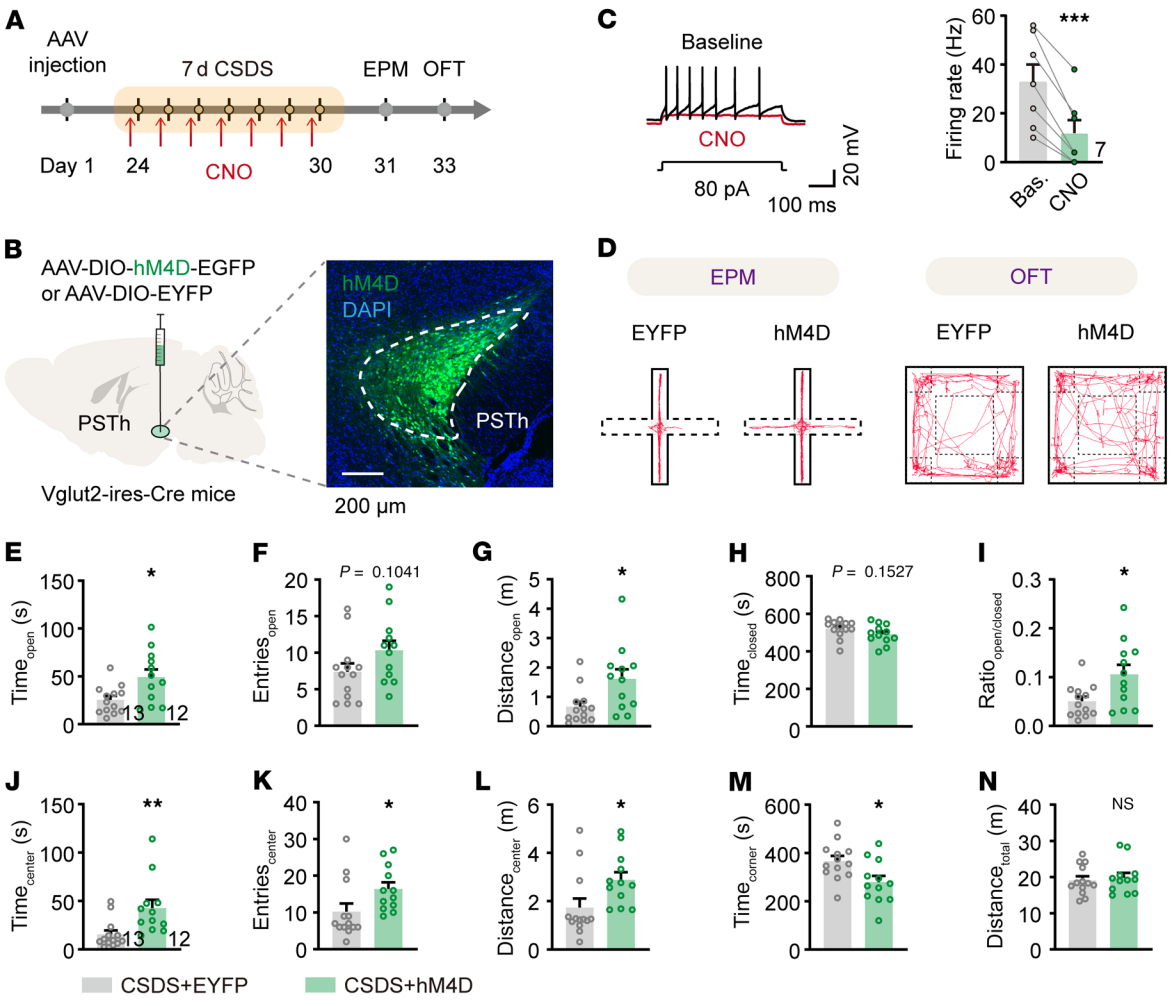
Inhibition of PSTh glutamatergic neurons during social defeat alleviates CSDS-induced anxiety-like behavior. (**A**) Experimental scheme showing pharmacogenetic inhibition of PSTh^Vglut2^ neurons during CSDS procedure and the measurement of anxiety-like behavior. (**B**) Schematic description and representative image of hM4D expression. Scale bar: 200 μm. (**C**) Raw traces (left) and statistical comparison of spike firing (right) in response to 80 pA current stimulus before and after CNO application (10 μM). *n* = 7 neurons from 2 mice. (**D**) Representative movement traces of an EYFP (left) and a hM4D mouse (right) in the EPM test or OFT after CSDS. (**E**–**I**) Behavioral statistics of EYFP and hM4D mice in the EPM test, including time spent (**E**), number of entries (**F**), distance traveled (**G**) in open arms, time spent in closed arms (**H**), and the open/closed ratio (**I**). (**J**–**N**) Behavioral statistics in the OFT, including time spent (**J**), number of entries (**K**), distance traveled (**L**) in the center zone, time spent in corner zones (**M**), and total distance traveled (**N**). *n* = 13 mice for the CSDS+EYFP group, and *n* = 12 mice for the CSDS+hM4D group. Data are shown as the mean ± SEM. *P < 0.05; **P < 0.01; ***P < 0.001; 2-tailed unpaired *t* test.

Since PSTh glutamatergic neurons were activated by acute stressors, including social defeat (Figure 1, E–G), we sought to determine whether the activity of these neurons during social defeat was necessary for anxiety-like behavior induced by CSDS. To test this hypothesis, we employed a pharmacogenetic approach to inhibit the activity of PSTh glutamatergic neurons during exposure to social defeat, followed by an assessment of anxiety-like behaviors using the EPM test and OFT after CSDS. Specifically, we bilaterally injected a Cre-dependent AAV vector encoding hM4D (AAV-DIO-hM4D-EGFP), a designer receptor exclusively activated by the ligand clozapine-*N*-oxide (CNO), into the PSTh of Vglut2-Cre mice. Control mice received the same virus vector carrying the fluorophore alone (AAV-DIO-EYFP) (Figure 2, A and B). Slice patch-clamp recordings demonstrated that administration of CNO (10 μM) hyperpolarized hM4D-expressed PSTh glutamatergic neurons and eliminated spiking induced by current injection, confirming the efficacy of the pharmacogenetic intervention (Figure 2C). Then, CNO (3 mg/kg) was administrated intraperitoneally 1 hour before social defeat for 7 consecutive days during CSDS. Behavioral results showed that the hM4D-infused CSDS group spent significantly more time exploring open arms of the EPM (Figure 2, E–I) and center zone of the open field (Figure 2, J–N) compared with the enhanced yellow fluorescent protein–infused (EYFP-infused) CSDS group, exhibiting an anxiolytic effect after chronic PSTh inhibition. Notably, 7 days of chronic inhibition of PSTh glutamatergic neurons did not alter basal anxiety-like behaviors in nonstressed control mice (Supplemental Figure 3, A–L). Additionally, to prevent a potential contamination effect from the last CNO infusion, we conducted an experiment in another cohort of mice with a 3-day washout period between the final CNO injection and behavioral tests. This experiment also showed significant anxiolytic effects in hM4D-injected versus EYFP-injected CSDS mice (Supplemental Figure 3, M–X). Together, these observations support that the activation of PSTh glutamatergic neurons is required for the induction of CSDS-induced anxiety-like behavior.

### The PSTh neurons remain hyperactive following CSDS.

**Figure 3:**
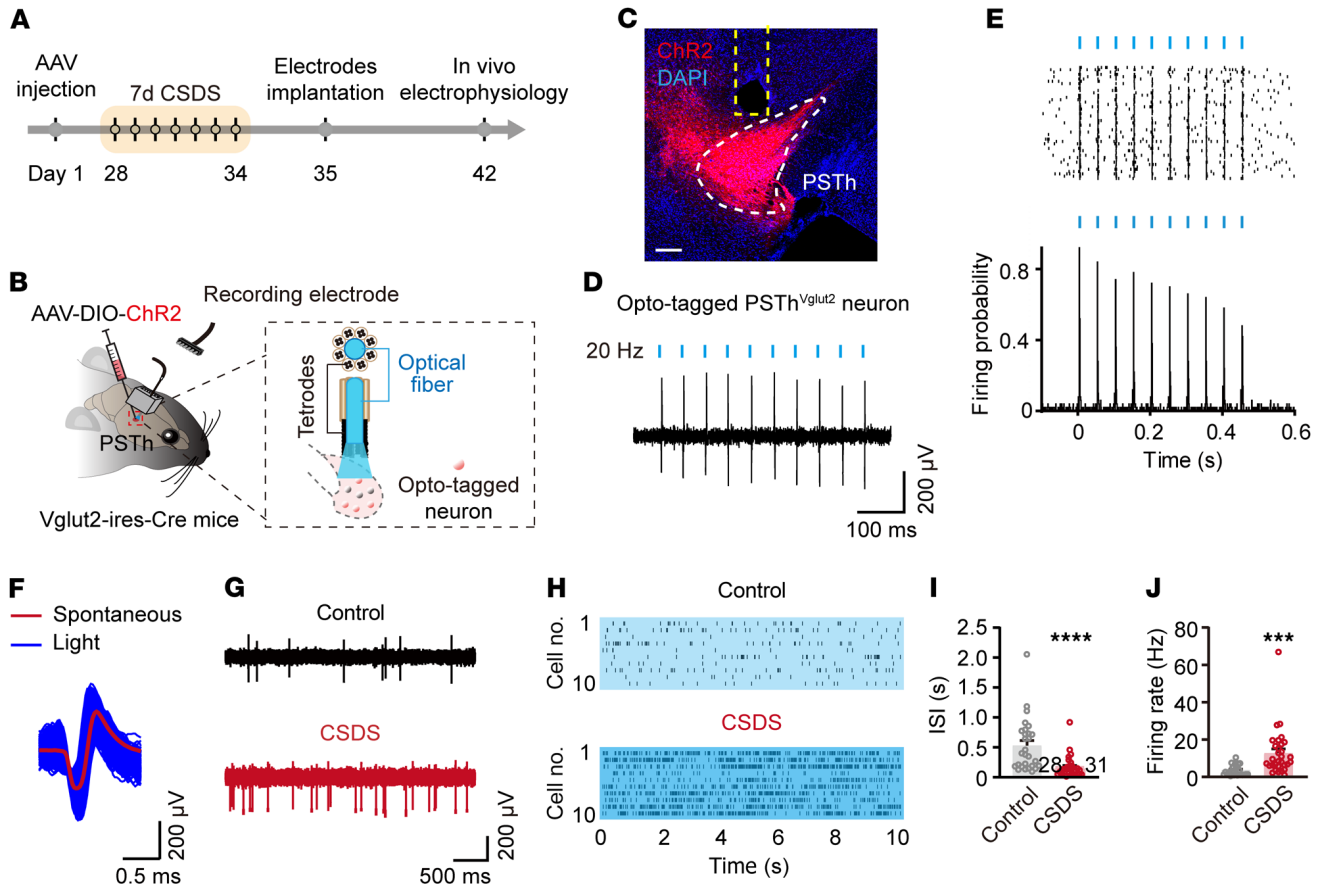
CSDS induces lasting hyperactivity in PSTh glutamatergic neurons. (**A** and **B**) Experimental timeline (**A**) and schematic diagram (**B**) of electrophysiological recordings of opto-tagged PSTh^Vglut2^ neurons after CSDS. (**C**) Example brain section showing ChR2 expression and optrode placement in the PSTh. Scale bar: 200 μm. (**D**) Representative trace of light-evoked spikes from an opto-tagged PSTh^Vglut2^ neuron. (**E**) Raster plots (top) and peristimulus spike time histogram (bottom) of multiple trials showing light-evoked spikes. (**F**) Averaged spontaneous (red) and light-evoked (blue) spike waveforms. (**G** and **H**) Representative traces (**G**) and raster plots (**H**) of opto-tagged PSTh^Vglut2^ neuronal spontaneous spikes. (**I** and **J**) The mean interspike interval (**I**) and spontaneous firing rate (**J**) of recorded PSTh^Vglut2^ neurons. *n* = 28 neurons from 4 mice for control, and *n* = 31 neurons from 7 mice for CSDS. Data are shown as the mean ± SEM. ***P < 0.001; ****P < 0.0001; 2-tailed unpaired *t* test.

Considering that the PSTh was activated by acute social defeat (Figure 1, E–G) and inhibition of PSTh glutamatergic neurons during social defeat reduced anxiety-like behavior (Figure 2 and Supplemental Figure 3, M–X), we sought to investigate the lasting impact of CSDS on PSTh neuronal activity. To address this, we recorded PSTh glutamatergic neurons using in vivo multiple-channel electrophysiology combined with an opto-tagging approach in freely moving animals (40, 41). Specifically, a Cre-dependent AAV carrying channelrhodopsin-2 (AAV-DIO-ChR2-mCherry) was unilaterally injected into the PSTh of Vglut2-Cre mice, and a customized mobile optrode, which consisted of 1 optical fiber surrounded by 8 tetrodes, was implanted in the PSTh (Figure 3, A–C). After a 5-minute baseline recording of spiking activity in the home cage of either CSDS or control mice, blue light pulses (473 nm, 20 Hz, ~0.2 mW, 1 ms pulse width) were delivered through the optical fiber. A single unit that exhibited a reliable light-evoked spike (spike probability of >90%) with short response latency (≤3 ms) was identified as Vglut2 neuron (control, *n* = 28; CSDS, *n* = 31) (Figure 3, D–F). We found that the interspike interval of opto-tagged glutamatergic neurons was significantly lower and their firing rate was significantly higher in CSDS mice relative to those in control mice (Figure 3, G–J). These results demonstrate that CSDS induces long-lasting hyperactivity in PSTh glutamatergic neurons.

**Figure 4:**
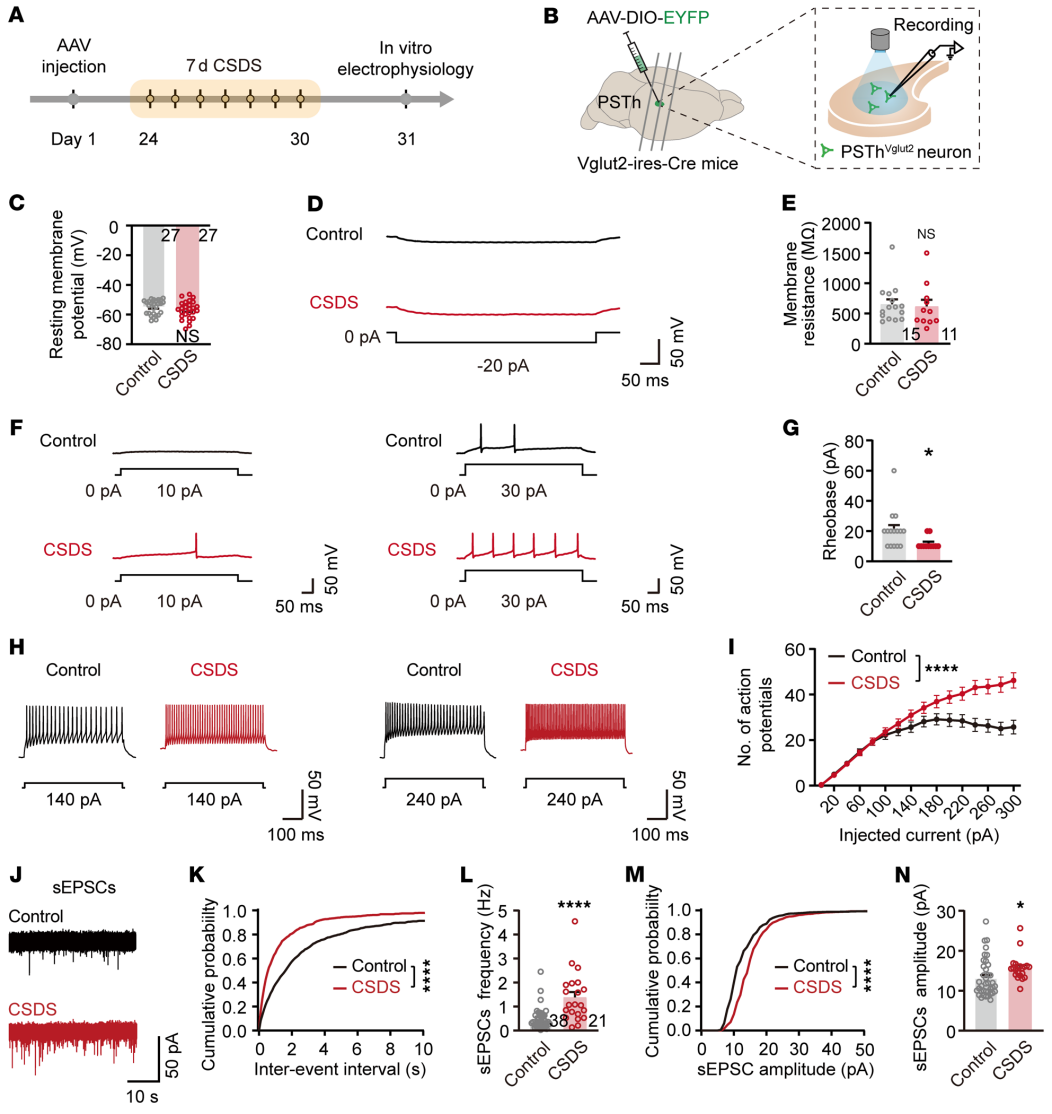
CSDS enhances the intrinsic excitability and excitatory synaptic inputs of PSTh glutamatergic neurons. (**A** and **B**) Experimental timeline (**A**) and schematic diagram (**B**) of whole-cell patch-clamp recordings of PSTh^Vglut2^ neurons after CSDS. (**C**) Comparison of resting membrane potentials between 2 groups. *n* = 27 cells from 5 mice for control, and *n* = 27 cells from 7 mice for CSDS. (**D** and **F**) Representative traces of different current injections. (**E** and **G**) Statistical comparison of the membrane resistance (**E**) and rheobase (**G**) of PSTh glutamatergic neurons between control and CSDS groups. *n* = 15 cells from 5 mice for control, and *n* = 11 cells from 7 mice for CSDS. (**H**) Representative traces of 140 pA (left) and 240 pA (right) current injections. (**I**) Number of action potentials in response to incremental current injections. (**J**) Representative recorded samples of PSTh^Vglut2^ neuronal sEPSCs. (**K** and **M**) Cumulative probability of interevent interval (**K**) and amplitude (**M**) of sEPSCs. (**L** and **N**) Comparison of sEPSC frequency (**L**) and amplitude (**N**) between 2 groups. *n* = 38 cells from 6 mice for control, and *n* =21 cells from 5 mice for CSDS. Data are shown as the mean ± SEM. *P < 0.05 and ****P < 0.0001; 2-tailed unpaired *t* test in **C**, **E**, **G**, **L**, and **N**; 2-way ANOVA, Bonferroni’s multiple-comparison post hoc tests in **I**, **K**, and **M**.

To dissect the underlying mechanisms of neuronal hyperactivity following CSDS, we analyzed cellular and synaptic properties of PSTh glutamatergic neurons using patch-clamp recordings in brain slices. Specifically, Vglut2-Cre mice were microinjected with a Cre-dependent AAV carrying EYFP to label PSTh glutamatergic neurons. Three weeks later, the mice underwent CSDS, and patch-clamp recordings were conducted in EYFP-positive cells from acute slices containing the PSTh (Figure 4, A and B). Under current-clamp mode, comparable resting membrane potentials and membrane resistance were observed, while decreased rheobase was observed following CSDS compared with nonstressed control mice (Figure 4, C–G). Furthermore, we observed a striking increase in the number of action potentials evoked by step current injections (500 ms, 0–300 pA, 20 pA steps) in the CSDS group (Figure 4, H and I), indicating that CSDS enhances the intrinsic excitability of PSTh glutamatergic neurons. To assess synaptic properties, we conducted voltage-clamp recordings with the membrane potential clamped at –70 mV. We found that both the frequencies and amplitudes of spontaneous excitatory postsynaptic currents (sEPSCs) were significantly increased in CSDS mice compared with controls (Figure 4, J–N). These findings suggest that CSDS enhances excitatory synaptic inputs onto PSTh glutamatergic neurons likely through both presynaptic and postsynaptic mechanisms.

Together, our data reveal that CSDS results in a lasting increase in the activity of PSTh glutamatergic neurons. Furthermore, this hyperactivity was attributable to enhanced intrinsic excitability and increased excitatory synaptic inputs.

### The PSTh is necessary for the expression of chronic stress–induced anxiety.

As mentioned above, CSDS induced anxiety-like behavior in mice (Supplemental Figure 2) and increased PSTh neuronal activity (Figures 3 and 4). This raised the intriguing question of whether there is a link between the PSTh neuronal hyperactivity and the animals’ anxiety-like behavior. To investigate this, we first artificially activated PSTh glutamatergic neurons and examined the consequence on anxiety-like behavior in naive mice. Specifically, we bilaterally expressed a Cre-dependent ChR2 vector (AAV-DIO-ChR2-mCherry) or a control vector (AAV-DIO-EYFP) in the PSTh of Vglut2-Cre mice. After 2 weeks of virus expression, optical fibers were implanted above the PSTh (Supplemental Figure 4, A and B). In vitro patch-clamp recordings confirmed that blue light pulses (473 nm, 20 Hz, 5 ms) effectively depolarized ChR2-expressing PSTh glutamatergic neurons and reliably evoked spikes (Supplemental Figure 4C). We then assessed animal behaviors using the EPM test and OFT, delivering blue light stimulation (~1 mW) during a 10-minute behavioral test. Compared with EYFP controls, ChR2-injected animals spent significantly less time in open arms and more time in closed arms of the EPM (Supplemental Figure 4, D–I). Similarly, ChR2-injected animals spent significantly less time in the center zone and slightly more time in corner zones of the OFT (Supplemental Figure 4, J–M). Additionally, reduced locomotion was evident in the OFT when PSTh glutamatergic neurons were optogenetically activated (Supplemental Figure 4N). Indeed, this suppression of locomotion was also observed in mice following CSDS (Supplemental Figure 2L) and is likely associated with an elevated anxious state (42). These observations demonstrate that optogenetic activation of PSTh glutamatergic neurons is sufficient to induce anxiety-like behavior in naive mice.

**Figure 5:**
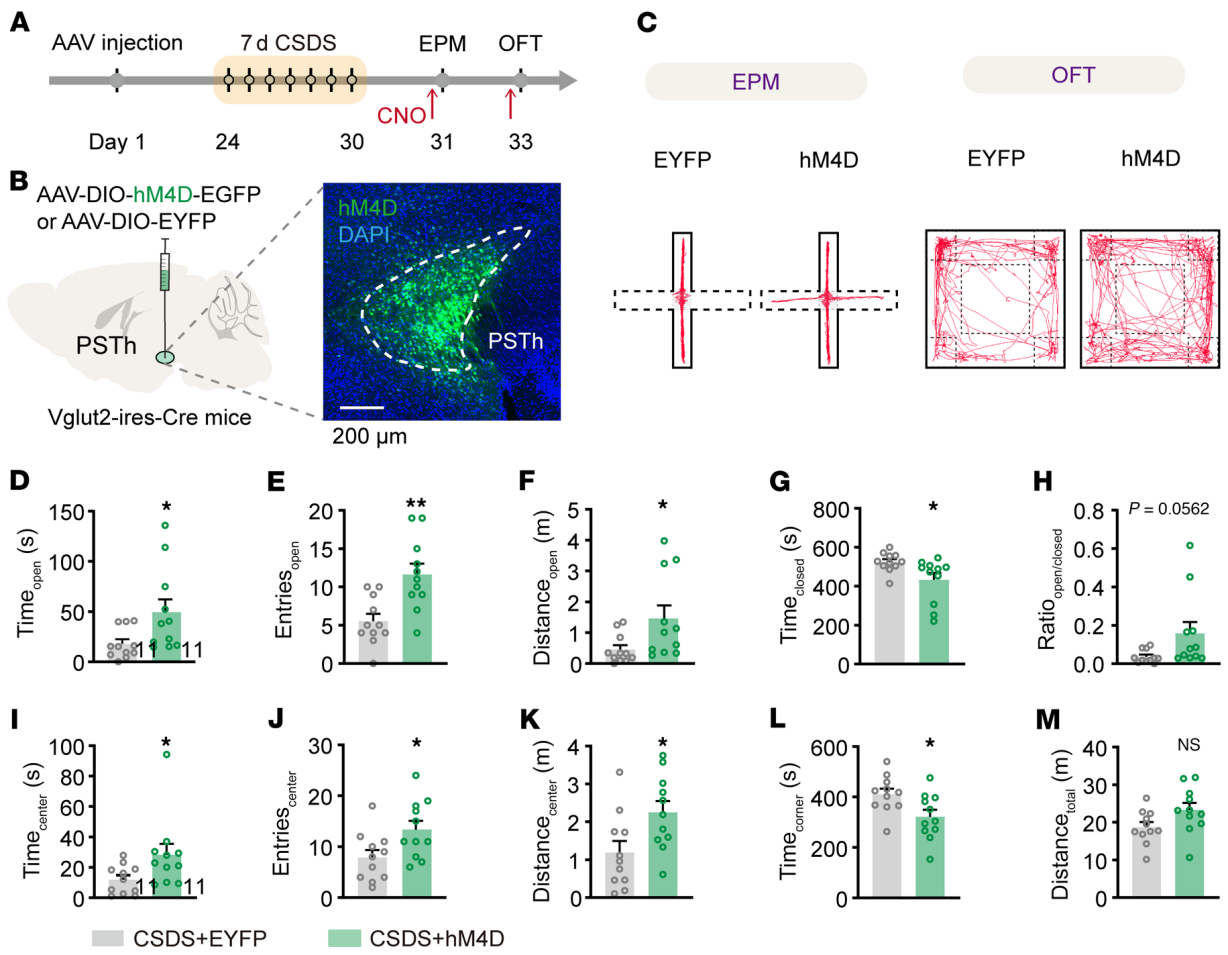
The activity of PSTh glutamatergic neurons is required for CSDS-induced anxiety-like behavior. (**A**) Experimental illustration showing pharmacogenetic manipulation during anxiety expression in CSDS mice. (**B**) Schematic description and representative image of hM4D expression. Scale bar: 200 μm. (**C**) Representative movement traces in the EPM test or OFT. (**D**–**H**) Behavioral statistics of the EPM test, including time spent (**D**), number of entries (**E**), distance traveled (**F**) in open arms, time spent in closed arms (**G**), and the open/closed ratio (**H**). (**I**–**M**) Behavioral statistics of the OFT, including time spent (**I**), number of entries (**J**), distance traveled (**K**) in the center zone, time spent in corner zones (**L**), and total distance traveled (**M**). *n* = 11 mice for each group. Data are shown as the mean ± SEM. *P < 0.05; **P < 0.01; 2-tailed unpaired *t* test.

Next, we further examined whether the hyperactivity of PSTh glutamatergic neurons following CSDS was necessary for the expression of CSDS-induced anxiety-like behavior. We employed a pharmacogenetic approach to suppress the activity of these neurons, administering CNO (3 mg/kg) intraperitoneally to CSDS mice 1 hour ahead of behavioral tests (Figure 5, A and B). When assessed using either the EPM test or OFT, inhibition of PSTh glutamatergic neurons significantly reduced anxiety-like behaviors in CSDS mice (Figure 5, C–M). Notably, acute inhibition of PSTh glutamatergic neurons did not affect locomotor activity or baseline anxiety-like behavior in naive mice (Supplemental Figure 5). Thus, the observed reduction in anxiety following PSTh inhibition in CSDS mice was not attributable to changes in baseline anxiety levels.

Taken together, these data demonstrate a causal role of PSTh in the expression of anxiety-like behavior induced by CSDS. On the one hand, activation of PSTh glutamatergic neurons was sufficient to induce anxiety-like behavior in naive mice (Supplemental Figure 4). On the other hand, inhibiting the hyperactivity of these neurons produced an anxiolytic effect in CSDS animals (Figure 5).

### The potentiated LPB-PSTh excitatory pathway mediates anxiety-like behavior.

To determine the neural circuitry underlying the role of PSTh glutamatergic neurons in anxiety-like behavior, we first explored their upstream inputs using a monosynaptic retrograde tracing technique with pseudotyped rabies virus (RV). In brief, Cre-dependent helper viruses (AAV-DIO-TVA-BFP and AAV-DIO-N2cG, 1:1 mixed) were unilaterally delivered into the PSTh of Vglut2-Cre mice. Three weeks later, RV (RV-CVS-EnvA-ΔG-tdTomato) was injected into the PSTh at the same coordinate (Supplemental Figure 6, A and B). After an additional week, the mice were euthanized, and retrogradely tdTomato-labeled neurons were observed in multiple brain regions, including the mPFC, BNST, CeA, PAG, and LPB (Supplemental Figure 6C). Notably, the LPB functions as a critical relay hub for ascending nociceptive information, representing a pivotal determinant of anxiety pathogenesis in CSDS paradigm (43–45). This region also plays a pivotal role in encoding aversive emotional state, with functional studies implicating its central involvement in fear and anxiety (42, 46). Given that LPB serves as an integration hub for both somatosensory and affective processing, we considered the LPB as a potential upstream input of PSTh-mediated anxiety. The LPB is rich in glutamatergic excitatory neurons (45). To further validate the LPB-PSTh glutamatergic connection, we infused mCherry-tagged ChR2 into the LPB of Vglut2-Cre mice and observed dense fluorescence-labeled axonal terminals throughout the entire PSTh (Supplemental Figure 6, D and E). Together, tracing studies indicated a robust glutamatergic projection from the LPB to the PSTh.

**Figure 6:**
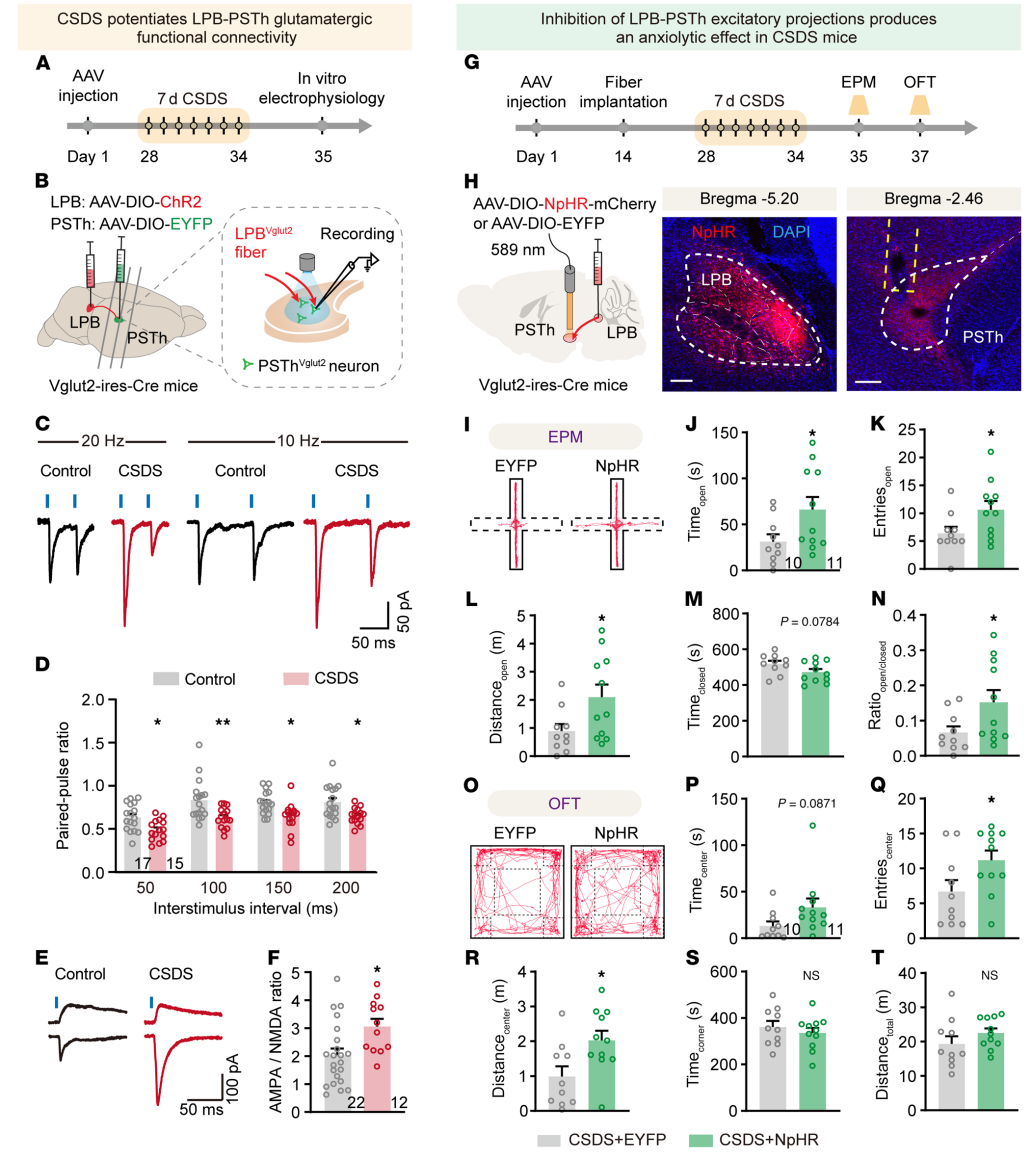
The potentiated LPB-PSTh excitatory pathway mediates anxiety-like behavior induced by CSDS. (**A** and **B**) Experimental timeline (**A**) and schematic diagram (**B**) of whole-cell patch-clamp recordings from LPB-PSTh projections. (**C**) Representative traces of postsynaptic currents recorded from PSTh^Vglut2^ neurons in response to paired-pulse light stimuli. (**D**) PPR of postsynaptic currents. *n* = 17 neurons from 7 mice for control, and *n* = 15 neurons from 3 mice for CSDS. (**E**) Representative traces of postsynaptic AMPA (bottom) and NMDA (top) currents. (**F**) Quantification of AMPA/NMDA ratio. *n* = 22 neurons from 7 mice for control, and *n* = 12 neurons from 3 mice for CSDS. (**G**) Experimental scheme showing optogenetic suppression of LPB-PSTh projections during anxiety expression in CSDS mice. (**H**) Schematic description and representative images of NpHR expression. Scale bars: 200 μm. (**I** and **O**) Representative movement traces after CSDS. (**J**–**N**) Behavioral statistics of the EPM test, including time spent (**J**), number of entries (**K**), distance traveled (**L**) in open arms, time spent in closed arms (**M**), and the open/closed ratio (**N**). (**P**–**T**) Behavioral statistics of the OFT, including time spent (**P**), number of entries (**Q**), distance traveled (**R**) in center zone, time spent in corner zones (**S**), and total distance traveled (**T**). *n* = 10 mice for the CSDS+EYFP group, and *n* = 11 mice for the CSDS+NpHR group. Data are shown as the mean ± SEM. *P < 0.05; **P < 0.01; 2-way ANOVA, Bonferroni’s multiple-comparison post hoc tests in **D**; 2-tailed unpaired *t* test in **F**, **J**–**N**, and **P**–**T**.

Employing an in vitro electrophysiological recording strategy, we next examined the functional connectivity of the LPB-PSTh glutamatergic pathway. We injected a Cre-dependent ChR2 vector (AAV-DIO-ChR2-mCherry) into the LPB and simultaneously expressed AAV-DIO-EYFP in the PSTh of Vglut2-Cre mice to label LPB and PSTh glutamatergic neurons, respectively. After 5 weeks of virus expression, we stimulated the ChR2-expressing axonal terminals of LPB glutamatergic neurons in the PSTh and simultaneously performed patch-clamp recordings from PSTh glutamatergic neurons in slice preparations (Figure 6B). We found that optical activation of LPB glutamatergic axonal terminals evoked excitatory postsynaptic currents (oEPSCs) in a large majority of PSTh glutamatergic neurons (95%, 19 out of 20 neurons) (Supplemental Figure 6F). Furthermore, these oEPSCs were blocked by tetrodotoxin (a sodium channel blocker) and augmented by 4-aminopyridine (a potassium channel blocker). Additionally, these oEPSCs were completely eliminated by glutamatergic receptor antagonists 6,7-dinitroquinoxaline-2,3(1H,4H)-dione (10 μM) and dl-2-amino-5-phosphonopentanoic acid (20 μM) (Supplemental Figure 6, G and H). These data confirm a monosynaptic functional connection between LPB glutamatergic neurons and PSTh glutamatergic neurons.

To determine the role of the LPB-PSTh excitatory pathway in CSDS-induced anxiety-like behavior, we next probed how this pathway responds to acute social stress using fiber photometry. Specifically, a retrograde virus carrying the Cre recombinase gene (AAV-Retro-Cre-mCherry) was unilaterally injected into the PSTh, and a Cre-dependent calcium indicator GCaMP6m vector (AAV-DIO-GCaMP6m) was delivered ipsilaterally into the LPB of WT C57 mice. An optical fiber was then implanted above the LPB (Supplemental Figure 7A). We observed a significant increase in fluorescence transients from PSTh-projecting LPB neurons each time the experimental mouse was attacked by an aggressive CD1 mouse (Supplemental Figure 7, B–E). These results indicate that the LPB-PSTh pathway is highly responsive to acute social stress.

Since the LPB-PSTh pathway is robustly activated by acute social defeat, we investigated whether CSDS had a lasting impact on the synaptic function of this glutamatergic pathway. We optically stimulated ChR2-expressing axon terminals of LPB glutamatergic neurons and recorded from PSTh glutamatergic neurons to assess the synaptic properties of LPB-PSTh glutamatergic projections (Figure 6, A and B). Our results showed that the paired-pulse ratio (PPR) of oEPSCs was significantly reduced in CSDS mice compared with controls (Figure 6, C and D), suggesting an increased probability of transmitter release from presynaptic LPB glutamatergic neurons. Moreover, the α-amino-3-hydroxy-5-methyl-4-isoxazole propionic acid (AMPA)/NMDA current ratio of the oEPSCs was elevated in CSDS mice (Figure 6, E and F), suggesting functional augmentation of postsynaptic AMPA receptors in PSTh glutamatergic neurons. Together, these data indicate that CSDS potentiates LPB-PSTh glutamatergic functional connectivity through both presynaptic and postsynaptic mechanisms.

Next, we directly evaluated the contribution of the potentiated LPB-PSTh glutamatergic pathway to anxiety-like behavior induced by CSDS. To do this, we bilaterally infused a Cre-dependent halorhodopsin (NpHR) vector (AAV-DIO-eNpHR3.0-mCherry) or an EYFP control vector (AAV-DIO-EYFP) into the LPB of Vglut2-Cre mice, followed by the implantation of optical fibers above the PSTh 2 weeks later (Figure 6, G and H). After 4 weeks of virus expression, both NpHR-expressing mice and EYFP control mice underwent CSDS. Behavioral tests were conducted the next day under continuous yellow light illumination (589 nm, ~5 mW) to specifically inhibit the LPB-PSTh glutamatergic pathway. Notably, the NpHR-expressing CSDS group spent significantly more time exploring open arms of the EPM and the center zone of the OFT compared with the EYFP-expressing CSDS group (Figure 6, I–T). These data indicate that the potentiated LPB-PSTh excitatory pathway is essential for the expression of anxiety-like behavior induced by CSDS. Since the LPB-PSTh projection was activated during acute social defeat (Supplemental Figure 7), we next investigated whether the projection is causally involved in anxiety induction. We expressed hM4D or EYFP in LPB and locally infused CNO into PSTh to silence the activity of the LPB-PSTh pathway during CSDS exposure (Supplemental Figure 8, A and B). Behavioral tests showed that chronic inhibition of the LPB-PSTh excitatory pathway produced an anxiolytic effect in both the EPM test and OFT (Supplemental Figure 8, C–N), confirming its necessity in anxiety induction. Furthermore, optical activation of the LPB-PSTh excitatory pathway elicited anxious state in naive mice, as assessed by the EPM test and OFT (Supplemental Figure 9). This suggests that activation of LPB-PSTh excitatory projections is sufficient to generate anxiety-like behavior.

Together, these findings demonstrate that potentiated LPB-PSTh glutamatergic transmission is pivotal to both anxiety induction and anxiety expression.

### The PSTh regulates anxiety-like behavior via its innervation upon the BNST.

To further explore the neuronal circuitry involving the PSTh in anxiety regulation, we exploited an anterograde tracing strategy to characterize the output targets of PSTh neurons receiving LPB inputs. Specifically, AAV1 was unilaterally delivered into the LPB to express Cre recombinase in an anterograde trans-synaptic manner, followed by infusion of a Cre-dependent EYFP vector (AAV-DIO-EYFP) into the PSTh (Supplemental Figure 10, A and B). Four weeks later, we euthanized mice and collected frozen brain sections (40 μm) for visualization under a microscope. We observed extensive EYFP-expressing axonal terminals in the striatum, septum, amygdala, thalamus, hypothalamus, and brainstem (Supplemental Figure 10, C–H), indicating a broad projection of PSTh neurons across the brain. Strikingly, the PSTh exhibited particularly strong projections to the CeA and BNST (Supplemental Figure 10, E and F). Besides, both CeA and BNST have well-established functions in regulation of stress and emotion (47, 48), and they are involved in mediating neurobiological consequences of chronic stress exposure, particularly through maladaptive plasticity that drives persistent functional dysregulation and affective disorder pathogenesis (24, 49). Thus, we prioritized these 2 targets for functional investigation. Consistently, anterograde tracing of PSTh glutamatergic neurons using AAV-FLEX-mGFP-2A-Synaptophysin-mRuby in Vglut2-Cre mice also revealed dense synaptophysin-mRuby terminals in the CeA and BNST (Supplemental Figure 10, I–L). These neuronal tracing studies provide anatomical evidence for the LPB-PSTh-CeA and LPB-PSTh-BNST excitatory trisynaptic circuits.

**Figure 7:**
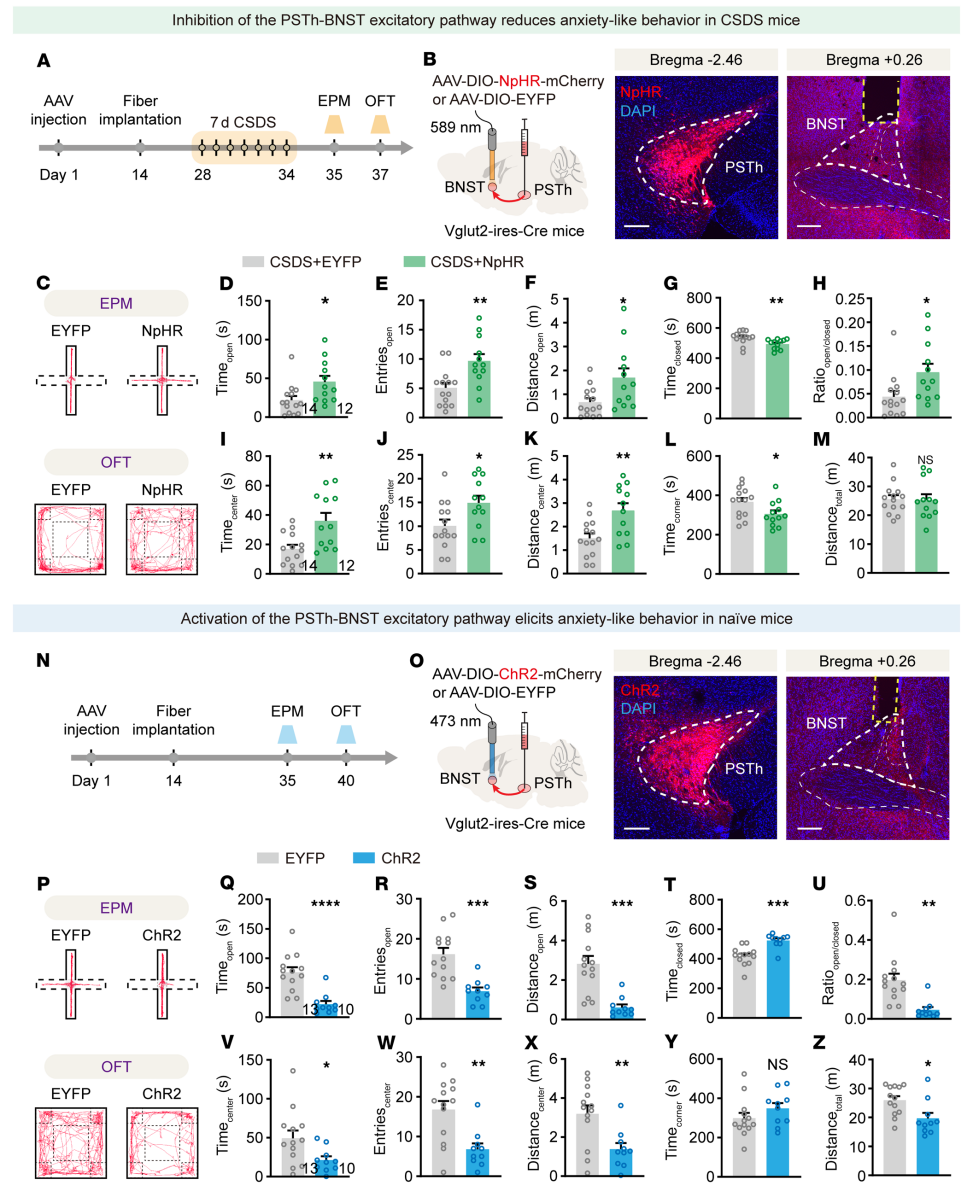
The PSTh regulates anxiety-like behavior via its innervation upon the BNST. (**A**) Experimental scheme showing optogenetic suppression of PSTh-BNST excitatory projections during anxiety expression in CSDS mice. (**B**) Schematic description and representative images of NpHR expression in PSTh glutamatergic neurons and axon terminals in BNST. Scale bars: 200 μm. (**C**) Representative movement traces of an EYFP (left) and a NpHR mouse (right) in the EPM test or OFT after CSDS. (**D**–**H**) Behavioral statistics of the EPM test, including time spent (**D**), number of entries (**E**), distance traveled (**F**) in open arms, time spent in closed arms (**G**), and the open/closed ratio (**H**). (**I**–**M**) Behavioral statistics of the OFT, including time spent (**I**), number of entries (**J**), distance traveled (**K**) in the center zone, time spent in corner zones (**L**), and total distance traveled (**M**). *n* = 14 mice for the CSDS+EYFP group, and *n* = 12 mice for the CSDS+NpHR group. (**N**) Experimental illustration showing optogenetic activation of the PSTh-BNST excitatory pathway during anxiety-like behavioral tests in naive mice. (**O**) Schematic description and representative images of ChR2 expression in PSTh glutamatergic neurons and axon terminals in BNST. Scale bars: 200 μm. (**P**–**Z**) The same as **C**–**M** but for EYFP- and ChR2-expressing naive mice. *n* = 13 mice for the EYFP group, and *n* = 10 mice for ChR2 group. Data are shown as the mean ± SEM. *P < 0.05; **P < 0.01; ***P < 0.001; ****P < 0.0001; 2-tailed, unpaired *t* test.

Next, we investigated whether the CeA and/or BNST serve as critical downstream target(s) of the PSTh in regulating CSDS-induced anxiety-like behavior. To do this, we infused mCherry-tagged NpHR bilaterally into the PSTh of Vglut2-Cre mice and implanted optical fibers in bilateral CeA or BNST to selectively inhibit PSTh-CeA or PSTh-BNST glutamatergic pathways in CSDS mice. We found that optogenetic inhibition of the PSTh-CeA glutamatergic pathway did not significantly affect anxiety-like behaviors (Supplemental Figure 11). In contrast, inhibiting the PSTh-BNST glutamatergic pathway resulted in a significant anxiolytic effect (Figure 7, A–M). Furthermore, direct optogenetic activation of the PSTh-BNST glutamatergic pathway elicited prominent anxiety-like behavior in unstressed naive mice (Figure 7, N–Z). Therefore, these results suggest that the BNST is a crucial target for PSTh glutamatergic neurons in their regulation of anxiety-like behavior induced by CSDS.

### Downregulated Kv4.3 in the PSTh is vital for anxiety-like behavior.

**Figure 8:**
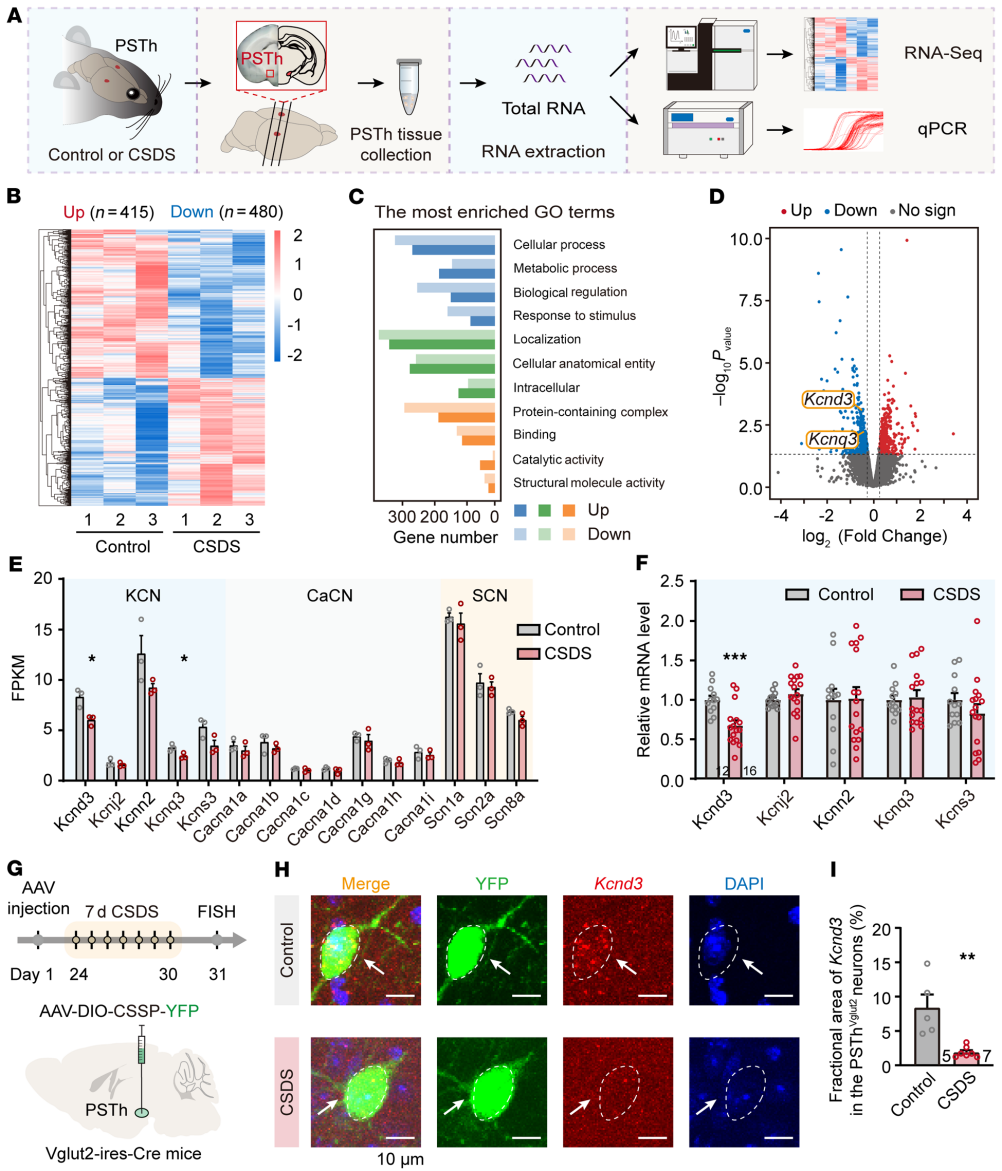
CSDS downregulates the expression of *Kcnd3* in PSTh glutamatergic neurons. (**A**) Schematic diagram for RNA-sequencing and qPCR measurements to examine the potential molecular mechanisms of PSTh in regulating anxiety-like behavior. (**B**–**D**) Heatmaps (**B**), the most enriched Gene Ontology terms (**C**), and volcano plot (**D**) of DEGs (*P* < 0.05 and fold change ≥ 1.2) in PSTh neurons from unstressed control and CSDS mice. Significantly upregulated genes are in red (*n* = 415), while downregulated genes are shown in blue (*n* = 480). Additionally, numerous genes show no significant differences between 2 groups (*n* = 13,359). (**E**) Normalized expression (fragments per kilobase of transcript per million fragments mapped; FPKM) of genes encoding important ion channels in PSTh neurons. *n* = 3 (30 mice in total) for each group. (**F**) qPCR results for *Kcnd3* level in PSTh neurons. *n* = 12 mice for the control, and *n* = 16 mice for the CSDS group. (**G**) Flow diagram and schematic illustration of virus injection for sparsely labeling PSTh^Vglut2^ neurons, enabling FISH verification of *Kcnd3* in these neurons. (**H**) Microscopy images show *Kcnd3* expression in PSTh^Vglut2^ neurons from a control (top) and a CSDS (bottom) mouse. Scale bars: 10 μm. (**I**) Quantification of the fractional area of *Kcnd3* in PSTh^Vglut2^ neurons from control and CSDS groups. *n* = 5 mice for control, and *n* = 7 mice for CSDS group. Data are shown as the mean ± SEM. *P < 0.05; **P < 0.01; ***P < 0.001; 2-tailed unpaired *t* test.

The aforementioned circuit dissection highlights the importance of the LPB-PSTh-BNST excitatory trisynaptic pathway in CSDS-induced anxiety. The lasting hyperactivity of PSTh neurons is the key for malfunction of this circuit and, hence, behavioral abnormality. It is therefore essential to uncover the molecular substrates responsible for the increased intrinsic excitability of PSTh neurons following CSDS (Figures 3 and 4). We postulated that transcriptional reprogramming mediated through differentially expressed genes (DEGs) might drive this electrophysiological phenotype. We therefore next conducted RNA sequencing experiments on isolated PSTh tissue (Figure 8A). The results identified 13,359 unchanged gene transcripts alongside 895 DEGs, with 415 transcripts upregulated and 480 transcripts downregulated in CSDS mice compared with controls (Figure 8, B–D). We then specifically focused on the DEGs related to ion channels, such as K^+^, Ca^2+^, and Na^+^ channel genes, all of which are key determinants of neuronal intrinsic excitability (50–52). Notably, there was a significant downregulation in *Kcnd3* and *Kcnq3* in the CSDS group, while Ca^2+^ and Na^+^ channel genes remained unchanged (Figure 8E). Quantitative real-time PCR (qPCR) further verified a decrease in *Kcnd3* transcripts of PSTh neurons from CSDS mice (Figure 8F). To confirm whether this reduction also occurred in PSTh glutamatergic neurons after CSDS, we delivered a Cre-dependent AAV vector encoding YFP (AAV-DIO-CSSP-YFP) into Vglut2-Cre mice to label these neurons. Using FISH staining, we observed a significant reduction in the relative abundance of *Kcnd3* staining in YFP-positive glutamatergic neurons following CSDS (Figure 8, G–I). Collectively, these findings indicate that CSDS disrupts *Kcnd3* gene transcription and downregulates Kv4.3 expression in PSTh glutamatergic neurons.

Given that Kv4.3 is essential for stabilizing membrane potential and preventing neuronal spiking (53–55), its downregulation could lead to overexcitability of PSTh glutamatergic neurons and contribute to anxiety-like behavior in CSDS mice. To test this hypothesis, we applied CRISPR-Cas9 gene-editing tools to selectively knock down Kv4.3 expression in these neurons (56). A Cre-inducible CRISPR-SaCas9 virus containing sgRNA targeting *Kcnd3* (AAV-DIO-SaCas9-sgKCND3) was infused into the PSTh of Vglut2-Cre mice (Supplemental Figure 12, A and B), resulting in increased action potentials in sgKCND3-expressing PSTh glutamatergic neurons compared with EYFP-expressing controls (Supplemental Figure 12C). Behavioral assays revealed that sgKCND3-induced Kv4.3 knockdown produced anxiogenic effects in unstressed naive mice (Supplemental Figure 12, D–N). These data indicate that Kv4.3 downregulation in PSTh glutamatergic neurons enhances their excitability and is sufficient to generate anxiety-like behavior.

**Figure 9:**
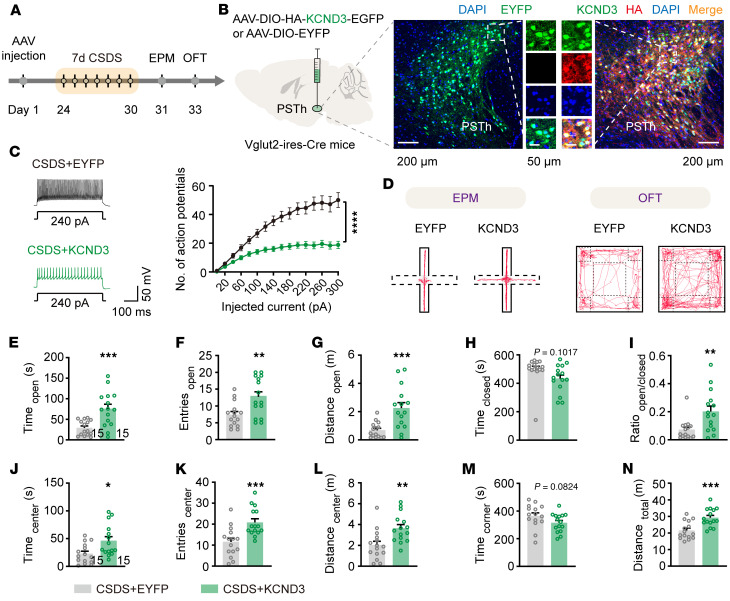
Kv4.3 replenishment in PSTh glutamatergic neurons rescues CSDS-induced anxiety-like behavior. (**A**) Experimental flowchart. (**B**) Schematic description and representative images of EYFP or KCND3-EGFP expression. Overlapping of KCND3-EGFP with HA suggests successful overexpression of *Kcnd3*. Scale bars: 200 μm, 50 μm (insets). (**C**) Representative traces (left) and statistical comparison of spike firing (right) of CSDS+EYFP- (dark) and CSDS+KCND3-expressed (green) PSTh^Vglut2^ neurons. *n* = 12 neurons from 3 mice for EYFP, and *n* = 26 neurons from 4 mice for KCND3. (**D**) Representative movement traces in the EPM test or OFT. (**E**–**I**) Behavioral statistics of the EPM test, including time spent (**E**), number of entries (**F**), distance traveled (**G**) in open arms, time spent in closed arms (**H**), and the open/closed ratio (**I**). (**J**–**N**) Behavioral statistics of the OFT, including time spent (**J**), number of entries (**K**), distance traveled (**L**) in the center zone, time spent in corner zones (**M**), and total distance traveled (**N**). *n* = 15 mice for each group. Data are shown as the mean ± SEM. *P < 0.05; **P < 0.01; ***P < 0.001; ****P < 0.0001; 2-way ANOVA, Bonferroni’s multiple-comparison post hoc tests in **C**; 2-tailed unpaired *t* test in **E**–**N**.

Next, we sought to determine if normalizing Kv4.3 expression could rescue anxiety-like behavior induced by CSDS. We bilaterally injected a Cre-dependent AAV encoding EGFP-tagged KCND3 (AAV-DIO-KCND3-EGFP) into the PSTh of Vglut2-Cre mice to replenish the expression of Kv4.3. A control virus carrying only EYFP (AAV-DIO-EYFP) was injected to serve as the control group (Figure 9, A and B). Notably, KCND3-expressing PSTh glutamatergic neurons fired much less action potentials than EYFP-expressing neurons in CSDS mice (Figure 9C). Behaviorally, KCND3-injected CSDS mice spent significantly more time exploring open arms and the central zone and exhibited more locomotion activity compared with the EYFP-injected CSDS mice (Figure 9, D–N). Taken together, these data demonstrate that downregulated Kv4.3 in the PSTh is essential for CSDS-induced anxiety-like behavior, and reexpression of these ion channels produces an anxiolytic effect.

## Discussion

Chronic stress represents one major etiological factor for anxiety pathology (33–35). Therefore, understanding how chronic stress is transformed into an anxiety state is of great importance. It has been proposed that anxiety is regulated by distributed brain structures and their intricate connections (6, 10, 57). Among these anxiety networks, the amygdala serves as a central hub for both induction and expression of stress-related anxiety (12, 22, 58). Specifically, the amygdala is initially activated by inputs from the LPB in response to stressful and noxious stimuli (44, 46, 59). This activation promotes the activity of the hypothalamus-pituitary-adrenal axis and the release of stress hormones (22, 34, 60). As a consequence of both neuronal and hormonal signals, chronic stress exposure results in lasting structural and functional alterations of the amygdala (22, 34, 61). The resulting hyperactivity of the amygdala ultimately manifests as behavioral expressions of stress-induced anxiety through its direct connections to efferent targets, including the BNST (23, 62). In addition to these classical findings, here we report a critical role of the PSTh in induction of CSDS-induced anxiety. In addition to the well-established LPB-amygdala-BNST circuitry, our research indicates that the LPB-PSTh-BNST excitatory trisynaptic pathway is also required for anxiety generation. Collectively, our study enriches existing anxiety networks centered on the amygdala and suggests a therapeutic target for disease prevention.

### PSTh neurons transform chronic stress into anxiety.

An anxiogenic stressor is a typical external aversive stimulus, while anxiety represents an internal emotional state. To investigate the neural substrates that link stressful stimuli to an anxious state is crucial for understanding anxiety generation. Previous studies suggested that brain structures directly activated by stressors can transform external stress into internal anxiety. For example, anxiogenic stressors elicit acute responses in excitatory neurons of the mPOA that mediate the expression of stress-induced anxiety-like behavior (11).

The PSTh is an understudied brain structure located dorsomedially adjacent to the caudal half of the subthalamic nucleus (STN) (63), and its physiological functions are still largely undefined. Recent studies indicated that the PSTh may serve as a hub for generating autonomic, emotional, and behavioral responses to interoceptive signals (28). For instance, PSTh neurons were activated by intraperitoneal infusion of LPS, Cisplatin, or Amylin (64, 65), and inhibiting PSTh neurons alleviated LPS-induced sickness (64) and anorexigenic hormone-induced appetite suppression (65). Furthermore, activation of PSTh neurons disturbed feeding behavior (29, 65). In this study, we demonstrate that PSTh neurons are also activated by various external stressors. First, in mice subjected to various stressors including social defeat, electric foot shock, and physical restraint, the number of c-Fos–positive neurons in the PSTh was significantly higher than that in unstressed control animals (Figure 1C). Additionally, when measured with fiber photometry in freely moving mice, we observed an instantaneous increase in neuronal activity of PSTh Vglut2 neurons in response to stressful stimuli (Figure 1, D–M). Notably, when PSTh Vglut2 neurons were inhibited during social defeat using pharmacogenetics, the animals subjected to CSDS exhibited significantly less anxiety-like behavior (Figure 2 and Supplemental Figure 3, M–X). Thus, PSTh glutamatergic neurons not only act as immediate neuronal effectors in response to stressful events, but they are also essential for initiating stress-induced anxious emotions.

Throughout an animal’s lifespan, the structure and function of the nervous system are constantly influenced by the external environment (66, 67). Strikingly, negative experiences, such as chronic stress, lead to pervasive alterations in neuronal excitability across anxiety networks, including the prefrontal cortex, amygdala, and ventral hippocampus (61, 68, 69). Similarly, functional changes in neuronal activity have been observed in PSTh glutamatergic neurons following CSDS. By combining in vivo multichannel recordings with opto-tagging techniques, we specifically recorded from PSTh Vglut2 neurons and found that CSDS induced hyperactivity in these neurons, as evidenced by significantly increased spontaneous firing rates (Figure 3). Patch-clamp recordings of brain slices further revealed that PSTh Vglut2 neurons in stressed animals received stronger excitatory synaptic inputs, as indicated by increased frequency and amplitude of their sEPSCs (Figure 4, J–N). In addition to synaptic alterations, PSTh Vglut2 neurons in the CSDS group exhibited increased intrinsic excitability, firing more action potentials than the control group in response to the same amounts of current injections (Figure 4, H and I).

Optogenetics provide an ideal tool for real-time manipulation of neuronal activity and have been widely used to dissect anxiety networks (12, 26, 70). In our study, we employed optogenetics and found that optogenetic activation of PSTh Vglut2 neurons can generate anxiety-like behavior in unstressed naive mice (Supplemental Figure 4). More importantly, inhibiting PSTh Vglut2 neurons reduced anxiety-like behavior in CSDS-exposed animals (Figure 5). Therefore, alterations in cellular and synaptic properties of PSTh neurons, along with their resultant hyperactivity following CSDS, significantly impacted the behavioral manifestation of anxiety. Together, our findings indicate that PSTh neurons are both necessary and sufficient for the initiation and maintenance of stress-induced anxiety-like behavior.

### LPB-PSTh-BNST trisynaptic circuit mediates chronic stress–induced anxiety.

Neurons perform their functions through an intricate network formed by their inputs and outputs. To understand how PSTh neurons were recruited by stressful stimuli, we conducted both anterograde and retrograde tracing studies. Consistent with previous reports (28, 31), we found that PSTh glutamatergic neurons received innervation from multiple brain regions, including the LPB (Supplemental Figure 6, A–E). In vitro patch-clamp recordings further confirmed that LPB glutamatergic neurons established monosynaptic functional connections with PSTh neurons (Supplemental Figure 6, F–H). Previous evidence suggests the LPB as a critical relay node that integrates nociceptive and affective pain components via spinoparabrachia-forebrain pathways (43) and mediates aversive emotional states, particularly anxiety and fear (32, 46). Given the LPB’s role in processing aversive sensory information and its monosynaptic connectivity to PSTh, this pathway likely serves as a critical drive of PSTh neuronal activation during chronic social stress. Indeed, we found that LPB neurons projecting to the PSTh were robustly activated when the experimental mouse was attacked by an aggressive mouse (Supplemental Figure 7). Therefore, the activation of the PSTh during social defeat can be partially attributed to excitatory inputs from the LPB.

Social defeat not only acutely activates the LPB-PSTh glutamatergic pathway but also modifies its efficacy. Following CSDS, the PPR and AMPA/NMDA current ratio of EPSCs recorded in PSTh neurons, stimulated optically via LPB glutamatergic axons, decreased and increased, respectively (Figure 6, A–F). These changes suggest that CSDS increases glutamate release probability in presynaptic LPB neurons and augmented AMPA receptor function in postsynaptic PSTh neurons. As a result of both presynaptic and postsynaptic plasticity changes, the strength of the LPB-PSTh glutamatergic transmission was potentiated after CSDS. Importantly, this potentiated pathway had a consequence on the animals’ behavior, as selective inhibition of the LPB-PSTh excitatory pathway alleviated anxiety-like behavior induced by CSDS (Figure 6, G–T, and Supplemental Figure 8). In fact, optogenetic activation of the LPB-PSTh excitatory projection elicited anxiety responses in unstressed naive mice (Supplemental Figure 9). Therefore, this pathway is both necessary and sufficient for anxiety generation. Together, LPB-PSTh glutamatergic projections likely provide a bottom-up route for conveying threatening information, contributing to the behavioral manifestations of anxiety.

PSTh neurons likely produce an anxiogenic effect through their innervation of brain structures within known anxiety networks. Consistent with previous findings, our tracing study revealed that PSTh glutamatergic neurons sent axonal fibers to several downstream targets (Supplemental Figure 10) (28, 31, 63). Among these efferent targets, the CeA and BNST have been extensively implicated in stress-induced behavioral dysregulation and affective disorders (25, 71). Intriguingly, inhibition of the PSTh-BNST glutamatergic pathway, but not the PSTh-CeA pathway, alleviated anxiety-like behavior in mice following CSDS (Figure 7, A–M, and Supplemental Figure 11). This pathway specificity may arise from the anatomical and functional distinctions between BNST- and CeA-projecting PSTh glutamatergic neurons.

Our anatomic tracing (Supplemental Figure 10) and functional analysis (Figures 6 and 7, and Supplemental Figure 8) together support the importance of the LPB-PSTh-BNST pathway in anxiety induction and expression. At the same time, it should be noted that the LPB and BNST are not the only input and output structures of the PSTh. Therefore, other upstream regions other than the LPB and downstream regions other than the BNST could also have a role in anxiety regulation. Further studies are needed to fully elucidate the pathway specificity of PSTh glutamatergic neurons in anxiety regulation.

### PSTh could be a therapeutic target for anxiety-related disorders.

Although anxiety disorders represent one of the largest health challenges in modern society, few novel therapeutics have emerged in recent decades. Clinically, deep brain stimulation (DBS) is used to treat tremors in Parkinson’s disease (PD), with electrodes implanted in the subthalamic area, which includes the STN, zona incerta, and PSTh (72–75). Beyond alleviating motor symptoms, PD patients also benefit from subthalamic DBS for nonmotor comorbidities, including emotional disturbances like anxiety (74, 76) and depression (72). Moreover, the improvement rate for emotional symptoms varies based on electrode locations and the volume of tissue stimulated within subthalamic areas among patients (73, 77). These observations suggest that different subnuclei of the subthalamic area may contribute differently to the improvement of anxiety symptoms in PD patients receiving subthalamic DBS, although the exact mechanisms remain unclear. Given the role of PSTh in controlling anxiety state, as demonstrated in the present study, subthalamic DBS could dampen PSTh hyperactivity and achieve an anxiolytic effect in PD patients. In this sense, the PSTh may serve as a specific neuromodulation target for anxiety treatment.

Voltage-gated K^+^ channels are widely expressed in the mammalian brain and play a crucial role in determining neuronal intrinsic excitability (50, 51). Recent studies have revealed that the regulation of gene expression of these channels in the whole brain or specific core areas is closely linked to anxiety-like behavior. For example, Kv1.3 knockout mice exhibited elevated anxiety-like behavior (78), while mice with depleted Kv4.1 in granule cells of the dentate gyrus displayed a reduced anxiety level (79). Notably, there is increasing evidence that alterations in the expression of voltage-gated K^+^ channels in various neuropsychiatric disorders contribute significantly to anxiety state associated with these diseases. For instance, decreases in Kv7.2 and Kv7.3 expression in a mouse model of autism spectrum disorders lead to abnormally increased excitability and firing rate of cortical neurons, contributing to disease-related anxiety (80). Conversely, enhancing Kv7.2 and Kv7.3 membrane protein expression in the lateral habenula reduces anxiety during alcohol withdrawal (81). In the present study, we discovered a significant reduction in Kv4.3 channel expression in the PSTh following CSDS at the mRNA level (Figure 8). Kv4.3 channels mediate subthreshold-operating A-type K^+^ currents (I_A_), which are essential for stabilizing neuronal membrane potential and preventing excessive spiking (53). Consistent with this, our results establish a pathological framework wherein chronic social stress enhances the excitability of PSTh neurons by reducing the expression of Kv4.3 channels, ultimately contributing to an internal anxiety state (Supplemental Figure 12). Furthermore, we found that reexpression of Kv4.3 in PSTh glutamatergic neurons dampened their hyperexcitability and alleviated anxiety-like behavior induced by CSDS (Figure 9). These findings suggest that pharmaceutical targeting of Kv4.3 channels in the PSTh may provide a promising avenue for anxiety intervention.

## Methods

Detailed information on materials and methods is provided in Supplemental Methods.

### Sex as a biological variable.

In this study, we utilized a well-established CSDS paradigm, which has been mostly established with males only, to investigate the mechanisms underlying transformation of chronic social stress into an anxiety state. Female mice exhibit periodic estrus, which induce cyclical fluctuations in key reproductive hormones and variations in physiological states that may influence behavioral outcomes. In contrast, male hormonal profiles maintain relative stability. The differing stress responses observed between sexes may be attributed to hormonal influences. Collectively, male subjects were selected to ensure consistency and repeatability of the experimental statistics in this study. Further research will include experiments with female mice to achieve a more comprehensive understanding.

### Animals.

Male C57BL/6 WT mice (Shanghai SLAC Laboratory Animal Co., Ltd.) and Vglut2-Cre mice (The Jackson Laboratory; strain 016963), aged 2–4 months, were used in this study. Retired breeder CD1 male mice (Beijing Vital River Laboratory Animal Technology Co., Ltd.), aged 8–10 months, served as aggressive social stressors. All animals were housed under a 12-hour light/dark cycle (lights on from 0700 to 1900 hours) at a stable temperature (22°C ± 1°C) and humidity (55% ± 5%), with food and water available ad libitum. Experimental mice were group housed (3 to 5 mice per cage), except for those in the CSDS paradigm or in vivo electrophysiological recordings. Each CSDS modeling mouse was cohoused with a CD1 mouse, separated by a perforated plastic divider, for 7 consecutive days, while control groups were physically separated from conspecifics during the same period. Animals were habituated to experimenters through gentle handling for at least 3 days prior to behavioral tests.

### Statistics.

Animal behaviors were video recorded and analyzed using the EthoVision XT video tracking system (Noldus). Brain slice patch-clamp recordings were conducted with pClamp 11 software (Molecular Devices), and analyzed with Clampfit (Molecular Devices) and Mini Analysis software (version 6.0.3; Synaptosoft Inc.). Statistical analyses for all immunohistochemical, behavioral, and electrophysiological experiments were performed using GraphPad Prism 8. MATLAB R2022b (MathWorks) was used to analyze calcium signals from fiber photometry tests. Generally, Student’s 2-tailed *t* test, 1-way ANOVA followed by Tukey’s posttests for multiple comparisons, or 2-way ANOVA followed by Bonferroni’s posttests for multiple comparisons was applied as appropriate. The number of animals used in each experiment and the statistical methods are indicated in the figure legends. All data are presented as mean ± SEM unless otherwise specified. A *P* value of less than 0.05 was considered statistically significant.

### Study approval.

Animal care and use were conducted in accordance with the guidelines approved by the IACUC of Zhejiang University in Hangzhou (ZJU20240879).

### Data availability.

The complete dataset generated in this study is described in the main paper or the supplemental material. Raw data are available in the Supporting Data Values file. RNA-sequencing data have been deposited in the NCBI Gene Expression Omnibus under accession number GSE298438.

## Author contributions

NL, JW, and HX conceived the project and designed the research. NL, JW, and HW performed all experiments and collected and analyzed data. NL was listed first as a co–first author due to responsibility for all experimental design, virus injection, behavioral tests, and immunofluorescence staining. JW conducted in vivo electrophysiological recordings and brain slice patch-clamp recordings. HW performed RNA sequencing, qPCR, and FISH experiments. BG, ZL, TLX, and SD provided resources and reviewed the manuscript. HX supervised the project and wrote the manuscript with contributions from all authors. All authors read and approved the manuscript.

## Supplementary Material

Supplemental data

Supporting data values

## Version 1 08/15/2025

Electronic publication
