# Supplementary material for: A noncanonical parasubthalamic nucleus–to–extended amygdala circuit converts chronic social stress into anxiety
Source: J Clin Invest. 2025 Aug 15;135(16):e188246. doi: 10.1172/JCI188246 (PMC12352892; doi:10.1172/JCI188246)
Supplement: Supplemental data [file jci-135-188246-s092.pdf]

A noncanonical parasubthalamic nucleus-to-extended amygdala circuit converts chronic social stress into anxiety

Authors

Na Liu,[1,2,3,4,#] Jun Wang,[1,2,3,4,#] Huan Wang,[1,#] Bin Gao,[1] Zheng Lin,[1] Tian-Le Xu,[5] Shumin Duan,[1,3,4] and Han Xu[1,2,3,4,6,*]

Affiliations

[1] Department of Psychiatry of the Second Affiliated Hospital and School of Brain Science and Brain Medicine, Zhejiang University School of Medicine, Hangzhou 310058, China.

[2] Nanhu Brain-computer Interface Institute, Hangzhou 311100, China.

[3] Liangzhu Laboratory, MOE Frontier Science Center for Brain Science and Brain-machine Integration, State Key Laboratory of Brain-machine Intelligence, Zhejiang University, 1369 West Wenyi Road, Hangzhou 311121, China.

[4] NHC and CAMS Key Laboratory of Medical Neurobiology, Zhejiang University, Hangzhou 310058, China.

[5] Collaborative Innovation Center for Brain Science, Department of Anatomy and Physiology, Shanghai Jiao Tong University School of Medicine, Shanghai 200023, China.

[6] Lingang Laboratory, Shanghai 200031, China.

Authorship note: Na Liu, Jun Wang and Huan Wang contributed equally to this work.

[*] Correspondence: E-mail: xuhan2014@zju.edu.cn

Conflict of interest: The authors have declared that no conflict of interest exists.

Supplemental Methods

Stereotactic surgeries, optical fiber and cannula implantation

GCaMP6m (AAV2/9-hSyn-DIO-GCaMP6m, 5.00 × 10^{12} genomic copies per mL), hM4D (AAV2/9-hSyn-DIO-hM4D(Gi)-EGFP, 2.62 × 10^{12} genomic copies per mL), ChR2 (AAV2/9-EF1a-double floxed-hChR2(H134R)-mCherry, 3.40 × 10^{12} genomic copies per mL), NpHR (AAV2/9-EF1a-DIO-eNpHR3.0-mCherry, 2.70 × 10^{12} genomic copies per mL), EYFP (AAV2/9-EF1a-DIO-EYFP, 1.46 × 10^{13} genomic copies per mL), TVA (AAV2/9-EF1a-DIO-H2B-BFP-T2A-TVA, 3.20 × 10^{12} genomic copies per mL), RVG (AAV2/9-Ef1α-DIO-N2cG, 5.16 × 10^{12} genomic copies per mL), RV (RV-CVS-EnvA-ΔG-tdTomato, 2.00 × 10^{8} integration units per mL), Retro-Cre (AAV2/2Retro-Plus-hSyn-Cre-mCherry, 1.59 × 10^{13} genomic copies per mL), AAV2/1-Cre (AAV2/1-hSyn-Cre-pA, 1.39 × 10^{13} genomic copies per mL), Synaptophysin (AAV2/9-hSyn-FLEx-mGFP-2A-Synaptophysin-mRuby, 1.55 × 10^{13} genomic copies per mL), CSSP (AAV2/9-hSyn-DIO-CSSP-YFP, 5.40 × 10^{12} genomic copies per mL), Cas9-sgKCND3 (AAV2/9-DIO-NLS-SaCas9-NLS-3×HA-bcH-polyA-U6-SasgRNA (KCND3), 5.01 × 10^{12} genomic copies per mL), KCND3 (AAV2/9-hSyn-DIO-3×HA-KCND3(human)-P2A-EGFP, 5.86 × 10^{12} genomic copies per mL) were produced by Taitool (Shanghai, China), BrainCase (Shenzhen, China) or BrainVTA (Wuhan, China).

All surgical experiments, including virus injection, fiber and cannula implantation, were performed under a microscope. Vglut2-Cre or C57BL/6 mice (8-9 weeks old) were anesthetized with isoflurane (4% for induction, 1% for maintenance) and

positioned in a stereotaxic frame (Stoelting Co., IL, USA) on a heating pad for maintaining body temperature. The skull was exposed, and a small craniotomy was perforated with a hand drill over the target region. Following coordinates relative to bregma were used for virus infusion (including PSTh, anterior-posterior (AP) -2.46 mm, medial-lateral (ML) ±1.00 mm, dorsal-ventral (DV) -4.75 mm; LPB, AP -5.20 mm, ML ±1.25 mm, DV -3.50 mm). Depending on the purpose of experiment, 50-200 nL of AAV virus was unilaterally or bilaterally injected into the target site using a syringe attached to a glass microelectrode (tip diameter 30 μm) controlled by an infusion pump (KD Scientific, USA) at a rate of 30 nL/min. The pipette was left in place for 10 minutes post-infusion to allow for virus diffusion.

For monitoring neuronal activity during fiber photometry tests, an optical fiber (200 μm O.D., 0.37 numerical aperture (NA), Inper Inc., Hangzhou, China), installed in a ceramic ferrule, was unilaterally inserted to target PSTh or LPB through the craniotomy following virus injection. Following coordinates relative to bregma were used for fiber implantation: PSTh (AP -2.46 mm, ML ±1.00 mm, DV -4.60 mm), LPB (AP -5.20 mm, ML ±1.25 mm, DV -3.40 mm). To record the firing activity of $PSTh^{Vglut2}$ neurons, customized movable optrodes (see **In vivo electrophysiological recordings** for details) were unilaterally implanted above PSTh (AP -2.46 mm, ML ±1.00 mm, DV -4.60 mm) on the day after CSDS or five weeks after virus injection for unstressed controls. In optogenetic manipulation experiments, optical fibers were bilaterally inserted into PSTh, CeA or BNST two weeks after virus injection using following coordinates: PSTh (AP -2.46 mm, ML ±1.60 mm, DV -4.60 mm with 6° angle), CeA

(AP -1.34 mm, ML ±2.50 mm, DV -4.25 mm), BNST (AP +0.26 mm, ML ±1.40 mm, DV -3.60 mm with 6° angle). To pharmacogenetic silence LPB-PSTh excitatory projections, cannulas (340 μm internal diameter, RWD) were bilaterally implanted above PSTh (AP -2.46 mm, ML ±1.10 mm, DV -4.60 mm) for drug infusion two weeks after virus injection. The ceramic ferrules were fastened on the skull using 3M Vetbond tissue adhesive and dental cement.

Behavioral experiments were conducted at least two weeks after virus delivery, fiber or cannula implantation to allow mice to recover.

CSDS paradigm

The CSDS paradigm performed in this study was modified from the conventional CSDS protocol (1). A customized two-chamber cage (30 cm long, 20 cm wide), separated by a vertically perforated plastic divider, was used to co-house an experimental mouse with an aggressive CD1 or a conspecific mouse. To execute the CSDS procedure, experimental mouse (intruder) was introduced into home cage of CD1 mouse (resident) and subjected to approximately three social defeat sessions (each bouts lasting 5-10 seconds) by the CD1 mouse (physical stress period). Following this, experimental mouse was separated from CD1 mouse by a divider for 24 hours, allowing continuous sensory exposure (visual, olfactory and auditory) without direct physical contact (sensory stress period). This procedure was repeated for seven consecutive days, using different CD1 residents as social stressors each day. In contrast, control animals were housed in pairs with an unfamiliar conspecific in the same cage to maintain normal

social conditions. Behavioral tests were conducted on day 1, 3, 4 or 6 following CSDS, during which all mice were single housed.

Behavioral procedures

All behavioral experiments were performed under dim light unless otherwise specified. Mice were allowed to acclimated to behavioral testing room for at least one hour prior to test. The overall activity of each mouse was automatically monitored by a video camera. The EthoVision XT video tracking system (Noldus, Netherland) was used to track location and movement of mice's head and body. After each behavioral session, apparatus was thoroughly cleaned with 75% ethanol to prevent olfactory cue bias.

Elevated Plus Maze (EPM) Test

A crossed maze with two enclosed arms and two opposing open arms (30×6.5 cm) was elevated 60 cm above the ground. Mice were softly placed in the center of maze (5×5 cm) facing open arm and allowed to move freely for a 10-minute session. Time spent, number of entries, and distance moved in open arms, as well as time spent in closed arms, were recorded and individually analyzed to assess anxiety-like behavior.

Open Field Test (OFT)

An enclosed acrylic open field arena (42×42×40 cm) was virtually divided into a center zone (21×21 cm) and a periphery area. Mice were gently placed in the peripheral area facing center zone and allowed to move freely for a 10-minute session. Time spent,

number of entries, and distance moved in the center zone, as well as time spent in four corner zones (9×9 cm), and total distance traveled were recorded and individually analyzed to measure anxiety-like behavior and locomotor activity.

Acute stressor exposure protocols for c-Fos mapping

To identify brain regions activated by acute stressors (social defeat, electrical shock, and physical restraint), we conducted whole-brain mapping for c-Fos protein expression. Experimental cohorts were subjected to the following stressor exposure protocols: social defeat mice experienced 10-minute social stress by CD1 intruder in their own home cages, while matched controls remained unstressed. Electrical shock groups received 10 times of random electrical stimuli (0.6 mA, 0.5 s) in shock apparatus, versus apparatus-exposed controls without shock. Restraint cohorts subjected 2-hour physical restraint, contrasted with freely-moving controls. All mice underwent transcardial perfusion 90 minutes post-stress for quantitative c-Fos expression.

Fiber photometry

To examine the activity of $PSTh^{Vglut2}$ neurons and LPB-PSTh pathway in response to stressful stimuli, a three-color multichannel fiber photometry system (ThinkerTech, Nanjing) was implemented to monitor calcium fluorescence signals via a GCaMP6m sensor (a genetically encoded calcium indicator). Specifically, calcium fluorescence fluctuations were obtained using a 470 nm blue light-emitting diode (LED), with a unique 405 nm reference signal employed to correct for movement artifacts. Calcium

transients were continuously collected in synchronization with animal behavior recordings by the EthoVision XT video tracking system during exposure to physical stressors.

For social defeat testing, an aggressive CD1 mouse was introduced into the home cage of experimental subject and typically initiated attacks within a few minutes. Trial onset was defined as the moment of attacks. In foot shock protocol, subject was placed in a standardized acrylic apparatus (20×20×40 cm) equipped with programmable grid floors delivering 10 times of random shocks (0.6 mA, 0.5 s duration). Stimulus onset corresponded to electrical shock generation. For air puff testing, subject received 10 times of air puff (1 s duration), with stimulus initiation marked by puff delivery. In forced swim testing, subject was positioned on an elevated platform that was randomly submerged 6 times (10 s duration). Trial onset was defined as the immersion moment.

Changes in calcium fluorescence (ΔF/F) were calculated using the formula $(F_{470}-F_0)/(F_0-F_{offset})$, where F_{offset} represents the averaged baseline fluorescence signals over 5 minutes during the dark condition before the optical fiber was attached to the implanted fiber on the mouse's skull. F_0 represents the fluorescence signals from a 2-second averaged baseline prior to introduction of stimuli. The maximal signal value within 2 seconds before stimuli was defined as the peak ΔF/F of baseline fluorescence signals, while the maximal signal value within 5 seconds after stimuli onset was defined as the peak ΔF/F of stressful event fluorescence signals.

In vivo electrophysiological recordings

The procedure of in vivo multi-channel electrophysiological recordings was carried out as previously described (2, 3). A custom-made movable optrode was inserted into PSTh of ChR2-expressed Vglut2-Cre mice, through a craniotomy. This optrode consisted of one optical fiber (O.D. 200 μm, NA 0.50; Inper Inc.) surrounded by eight tetrodes, with the tips of microwires protruding approximately 300 μm beyond the fiber. Each tetrode was made of four twisted fine platinum/iridium microwires (12.5 μm diameter, California Fine Wire), which were electroplated with gold to achieve a final impedance of 300-400 kΩ by a NanoZ impedance testing system (Plexon Inc., USA). Following a seven-day recovery from electrode implantation, mice were acclimatized to a headstage (Plexon Inc., USA) and connecting cables attached to the electrodes on their skull for at least three days before formal electrophysiological recordings. To investigate the spiking activity of $PSTh^{Vglut2}$ neurons after CSDS, multichannel electrical signals were recorded from awake and calm mice in their home cages. Recording began with soft connections to the headstage, and nitrogen balloon were used to suspend cables, allowing free movement. Behavior monitoring was conducted simultaneously with a CinePlex behavioral research system (Plexon Inc., USA). Spiking activities were digitized at 40 kHz, bandpass filtered between 250-8000 Hz, and stored on a PC for further offline analysis. Specifically, a 5-minute recording of spontaneous multi-unit firing activity of PSTh neurons was conducted from both CSDS and control mice. To electrophysiological identify $PSTh^{Vglut2}$ neurons, 50 strings of 10 blue light pulse (473 nm, 20 Hz, 1 ms duration, ~0.2 mW at fiber tip) were delivered at 20 s intervals. Units were classified as directly light-responsive if they exhibited time-locked spiking with

high reliability (> 90%), short first-spike latency (≤ 3 ms) in response to light stimulation, and high correlation coefficient between photo-evoked and spontaneous waveform (> 0.9). The optrode depth was gradually adjusted by ~40 μm daily to record as many units as possible. At the end of the last recording session, a small current (30 μA, 20 s) was applied to electrodes to destroy the surrounding brain tissue. Subsequently, PFA perfusion was performed to verify the location of ChR2 expression and electrode tips in the PSTh.

Brain slice patch clamp recordings

To determine the electrophysiological properties of $PSTh^{Vglut2}$ neurons (EYFP-tagged) and the functional connectivity in LPB^{Vglut2}-$PSTh^{Vglut2}$ projections (ChR2-expressed LPB^{Vglut2} neurons and EYFP-labeled $PSTh^{Vglut2}$ neurons) in both CSDS and unstressed control mice, in vitro whole-cell clamp patch recordings were carried out on acute coronal brain slices according to previously protocols (4). Specially, animals were first anesthetized and then transcardially perfused with 20 mL of cold modified artificial cerebrospinal fluid (ACSF) containing (in mM): 87 NaCl, 2.5 KCl, 1.25 NaH_2PO_4, 26 $NaHCO_3$, 1 $CaCl_2 \cdot 2H_2O$, 2 $MgSO_4 \cdot 7H_2O$, 75 sucrose and 10 glucose (oxygenated with 95% O_2 and 5% CO_2, pH 7.40, 300-305 mOsm). Acute coronal slices at 260 μm thickness containing the PSTh region were sectioned using a vibrating microtome (Leica VT1200S, Germany) and quickly transferred into warm (32 °C) and oxygenated modified ACSF to recover for 30 minutes. Then, slices were maintained in standard ACSF (composition in mM: 119 NaCl, 2.5 KCl, 1.25 NaH_2PO_4, 24 $NaHCO_3$, 2

$CaCl_2 \cdot 2H_2O$、2 $MgSO_4 \cdot 7H_2O$ and 12.5 glucose saturated with 95% O_2 and 5% CO_2, pH 7.40, 300-305 mOsm) for at least 1 hour at room temperature. For recording, collected PSTh sections were transferred into a recording chamber (Warner Instruments, USA) and continuously perfused with standard ACSF equilibrated with 95% O_2 and 5% CO_2 (~2 mL/min) at 30-32 °C. Patch recording pipettes (5-8 MΩ) were filled with an internal solution and connected to a MultiClamp 700B amplifier (Molecular Devices, USA) and a 1440A Digitizer Acquisition System (Molecular Devices, USA). For current-clamp recordings, the patch electrodes were filled with internal solution (composition in mM: 130 potassium gluconate, 6.3 KCl, 0.5 EGTA, 10 HEPES, 4 Mg-ATP, 0.3 Na-GTP and 5 phosphocreatine; pH, 7.2-7.3; osmolarity, 285-290 mOsm). For voltage-clamp recordings, the patch electrodes were filled with internal solution (composition in mM: 110 $CsMeSO_3$, 20 TEA-Cl, 15 CsCl, 0.5 EGTA, 10 HEPES, 4 Mg-ATP, 0.3 Na-GTP, 4 QX-314, and 1 spermine; pH, 7.2-7.3; osmolarity, 285-290 mOsm/kg). Electrophysiological signals were low-pass filtered at 5 kHz and digitally samples at 10 kHz on-line during recordings, with subsequent offline analysis conducted using pClamp 11 software (Molecular Devices). Only cells exhibiting stable series resistance (less than 20% change) throughout the recording period were included for further analysis. All chemicals used in brain slice recording experiments were purchased from Sigma, unless otherwise specified.

To examine electrophysiological properties of $PSTh^{Vglut2}$ neurons after CSDS, spontaneous activity was monitored in current-clamp mode after stabilizing the recordings. Intrinsic excitability was assessed by injecting steady-state currents in 20

pA increments from 0 to 300 pA, using synaptic blockers 6,7-dinitroquinoxaline-2,3-dione (DNQX, 10 μM, Sigma-Aldrich), DL-2-Amino-5-phosphonopentanoic acid (AP-5, 20 μM, Sigma-Aldrich) and gabazine (10 μM, Sigma-Aldrich) to block synaptic transmissions. Rheobases were determined as the minimal current step required to trigger an action potential. Membrane resistance was calculated as the change in voltage elicited by -20 pA hyperpolarizing current injection. To avoid influence by spontaneous action potentials, rheobases and membrane resistance were computed for silent neurons only. Spontaneous excitatory post-synaptic currents (sEPSCs) of $PSTh^{Vglut2}$ neurons were recorded in voltage-clamp mode at -70 mV while blocking inhibitory postsynaptic currents with gabazine (10 μM) in standard ACSF.

To explore efficiency of synaptic transmission in LPB^{Vglut2}-$PSTh^{Vglut2}$ projections, cells were clamped at -70 mV in a voltage-clamp mode and blue light pulses (470 nm, ~1 mV, 1 ms pulse width) generated by LED (Mightex, Toronto, Canada) were delivered every 20 s to stimulate the axon terminals of LPB^{Vglut2} neurons in PSTh. Tetrodotoxin (TTX, 1 μM, Refinebio Inc., Dalian, China) and 4-aminopyridine (4-AP, 100 μM, Sigma-Aldrich) were added for verifying monosynaptic responses evoked by blue light pulse. DNQX (10 μM) and AP-5 (20 μM) were bath administrated to determine excitatory nature of LPB^{Vglut2}-$PSTh^{Vglut2}$ projections.

To evaluate synaptic release probability, the paired-pulse ratio (PPR) of EPSCs was measured using varying intervals (50, 100, 150, and 200 ms) between blue light pulses (470 nm, 1 ms pulse width). The PPR was calculated by dividing the amplitude of the second EPSC by that of the first. To detect postsynaptic receptors-mediated

current, cells were initially held at -70 mV in a voltage-clamp mode to record AMPA current, and the holding potential was then gradually increased to +40 mV to record NMDA current. The amplitude of NMDA current was analyzed 50 ms after light stimulation to minimize the influence of AMPA currents.

To verify the efficacy of ChR2, hM4D, sgKCND3 and KCND3, PSTh coronal slices were prepared similarly as abovementioned. Then, whole-cell current-clamp recordings were performed from $PSTh^{Vglut2}$ neurons infected with ChR2, hM4D, sgKCND3 or KCND3. Blue light pulses at 20 Hz were applied to assess spike responses in ChR2-expressed neurons. For hM4D-injected neurons, spontaneous spikes were recorded both before and after CNO administration (10 μM). For neurons infused with sgKCND3 or KCND3, spontaneous activities were measured in PSTh slices to evaluate their effects.

Pharmacogenetic manipulation

For hM4D-mediated pharmacogenetic inactivation of $PSTh^{Vglut2}$ neurons, Clozapine-N-Oxide (CNO, C0832, Sigma) was dissolved in saline (0.9% NaCl solution) to achieve a working concentration of 0.3 mg/mL. CNO was administered intraperitoneally to hM4D-expressed mice at a dosage of 3 mg/kg body weight one hour prior to social defeat exposure or behavioral experiments (5). The same amount and timeline of CNO injection were also applied to EYFP-infused control mice to prevent from potential confounding effect of CNO metabolite. For hM4D-mediated suppression of LPB-PSTh excitatory projections, CNO (5 μM in 120 nL) was locally infused into PSTh through

the stereotaxically implanted guide cannula and then waited a few minutes to facilitate localized drug diffusion. Mice were exposed to social stress 30 minutes following CNO infusion. The EYFP control group received the same regimen.

Optogenetic manipulation

In optogenetic experiments, optical fibers were attached to a laser generator (Inper Inc.) through a dual optical fiber patch cord. The laser beam (473 nm for activation or 589 nm for inhibition) was split into two beams through a commutator and connected with the implanted optical fibers to specifically optogenetic activation or inhibition of neurons and terminals (6). All opto-modulation experiments were performed while the mice behaviors were being video-tracked. For optogenetic activation of $PSTh^{Vglut2}$ neurons and their projections, a 473 nm blue laser pulsed at 20 Hz, 5 ms, and 0.5-1.0 mW was employed in ChR2-injected mice during 10-minute behavioral tests (7). Conversely, a 589 nm yellow laser was continuously delivered at 5 mW during 10-minute tests to inhibit projections in NpHR-injected mice. Control mice infused with EYFP underwent the same opto-stimulation to prevent any confounding effect from laser illumination.

Immunofluorescence staining

Mice were anesthetized and underwent transcardial perfusion with 40 mL phosphate-buffered saline (PBS) followed by 40 mL of ice-cold 4% paraformaldehyde (PFA) solution. After brain extraction, the tissue was postfixed in 4% PFA overnight at 4 °C

and dehydrated in 30% sucrose solution until it sank to the bottom. Brain tissues were then embedded in Tissure-Tek O.C.T. Compound (Sakura, Japan) and sliced into 40 μm coronal sections using a cryostat (Leica CM1950). Free-floating slices were rinsed with PBS three times for 10 min each on a shaker. For immunofluorescence experiments, 40 μm sections were permeabilized with 0.3% Triton X-100, and blocked in PBS containing 10% normal goat serum (NGS) and 0.1% bovine serum albumin (BSA) for 1.5 h at room temperature. Brain slices were sequentially incubated overnight with primary antibodies diluted with 0.1% NGS, 0.1% BSA and 0.3% Triton X-100 in PBS at 4 °C. The following primary antibodies were used: rabbit anti-cFos (1:1000; Cell Signaling Technology, 2250), rabbit anti-GFP (1:1000; Abcam, ab6556) and mouse anti-HA (1:1000; CST, 2367). After washed three times with PBS for 15 min each, brain slices were then incubated with secondary anti-bodies in diluted blocking solution for 2 h at room temperature in darkness. The secondary antibodies used were goat anti-rabbit antibodies conjugated to Alexa Fluor 488 (1:1000; Abcam, ab150077) or goat anti-mouse antibodies conjugated to Alexa Fluor 647 (1:1000; Abcam, ab150115). Then, slices were washed four times with PBS for 15 min each. After all aforementioned procedures, nuclei were stained with DAPI, and finally washed 5 min with PBS. Finally, slices were mounted on microscopy slides under glass coverslips with mounting media. Confocal images were acquired with a confocal microscope (Olympus, FV1200) with a 10×, 20×, or 40× objective. ImageJ was executed to quantify c-Fos positive cells.

RNA sequencing protocols

RNA sequencing technique was introduced to identify differentially expressed genes (DEGs) within the PSTh following CSDS (8). CSDS-experienced mice or unstressed control mice were transcardially perfused with RNA-free PBS. The brains were quickly frozen in liquid nitrogen, and PSTh tissues were collected under a dissecting microscope. **RNA extraction**: Total RNA was extracted from PSTh tissues using TRIzol Reagent (Life Technologies, CA, USA). **RNA quantification and qualification**: The concentration and purity of RNA samples were ensured using NanoDrop 2000 (Thermo Fisher Scientific, Wilmington, DE). The number of RNA integrity, which was assessed via the Agilent Bioanalyzer 2100 system (Agilent Technologies, CA, USA), should be ≥7 to allowed subsequent analysis. **Library preparation**: Sequencing libraries were generated using Hieff NGS Ultima Dual-mode mRNA Library Prep Kit for Illumina (Yeasen Biotechnology Co., Ltd., Shanghai, China) according to manufacturer's protocol and index codes were added to attribute sequences to each sample. **Sequence**: The libraries were sequenced on the Illumina NovaSeq platform to generate 150 bp paired-end reads following the manufacturer's procedure. **Quality control**: Clean reads were obtained by removing reads containing adapter, ploy-N and low-quality reads from raw data. **Reads mapping to the reference genome**: Clean reads were mapped to the reference genome sequence using Hisat2 tools soft. Only reads with a perfect match or one mismatch were further analyzed and annotated based on the reference genome. **Quantification of gene expression levels**:

Gene expression levels were measured by fragments per kilobase of transcript per million fragments mapped (FPKM). The formula for FPKM is shown below:

$$\text{FPKM} = \frac{\text{cDNA Fragments}}{\text{Mapped Fragments (Millions)} * \text{Transcript Length (kb)}}$$

Differential expression analysis: The genes, which expressed significantly different from unstressed vs CSDS mice by DESeq2 processing, were defined as Differentially Expressed Genes (DEGs). Criteria for DEGs was set as Fold Change (FC) ≥ 1.2 and P-value < 0.05. **GO enrichment analysis**: Gene Ontology (GO) enrichment analysis of DEGs was implemented by the clusterProfiler R packages (Bioconductor, TU Dortmund, Germany).

Reverse transcription and qPCR

qPCR was implemented to verify RNA sequencing results as described previously (9). Total mRNA was extracted from PSTh tissues of CSDS or control groups using the SteadyPure Universal RNA Extraction Kit (Accurate Biology, AG21017, China). Then, the mRNA was reverse transcribed to produce cDNA using the Evo M-MLV RT Mix Kit (Accurate Biology, AG11728, China), followed by amplification through PCR procedure. qPCR was conducted to analyze the mRNA level using SYBR Green (Accurate Biology, AG 11701, China) on a Light Cycler 480 platform (Roche, Basel, Switzerland). The relative mRNA expression levels were quantified and normalized to the calibrator gene *Gapdh* by employing the 2-ΔΔCt method. The specific primers for qPCR were as follows:

Kcnd3 primer (F): AGGGCACAAAGAAGGCCC

Kcnd3 primer (R): TAAGCAGTGTAGCAGGTGGTG

Kcnj2 primer (F): TGCGTGTCAGAGGTCAACAG

Kcnj2 primer (R): TGCAGCCTACGATTGACTGG

Kcnn2 primer (F): GGAGTCTGCTTGCTTACTGGA

Kcnn2 primer (R): CGGCTGCGTTTTTCACTCTTT

*kcnq3*primer (F): TGTCTCTTTCCCATTCTTCAGGAAA

Kcnq3 primer (R): AAGGCGAACCCGATCCAAGA

Kcns3 primer (F): CTACACGCTGCCCTTGCATA

Kcns3 primer (R): GGAGTCACCGAGAAATGGCT

Gapdh primer (F): AAATGGTGAAGGTCGGTGTGAAC

Gapdh primer (R): AAATGGTGAAGGTCGGTGTGAAC

Fluorescent RNAscope in situ hybridization

To examine *Kcnd3* expression in $PSTh^{Vglut2}$ neurons of CSDS-exposed and unstressed groups, the RNAscope Fresh Frozen Multiplex Fluorescent kit (ACD, 323100) and immunolabeling were used on PSTh brain slices expressing DIO-CSSP-YFP (9). All procedures were conducted in an RNA-free environment to prevent RNA degradation. Following post-fixation and dehydration, coronal PSTh slices were sectioned coronally at 20 µm thickness and stored at -20°C until further analysis. The RNAscope assay was performed adhering to the manufacturer's protocol. The sections were dehydrated through a graded ethanol series (50%, 70%, and twice in 100%) before incubation with Proteinase IV for 30 minutes at room temperature. Hybridization and signal

amplification were performed using probes targeting *Kcnd3* (Advanced Cell Diagnostics, CA, USA) in a hybridization oven set at 40°C. Detection of *Kcnd3* signals were tagged with Opal 520 dye (1:2500, PerkinElmer). After completion of RNAscope for *Kcnd3*, GFP staining was conducted to amplify fluorescence signals from DIO-CSSP-YFP. Finally, nuclei were counterstained with DAPI for 30 seconds at room temperature, and sections were mounted with Prolong Gold antifade reagent (Thermo Fisher Scientific, Waltham, Massachusetts, USA).

Reference

1. Golden SA, et al. A standardized protocol for repeated social defeat stress in mice. *Nat Protoc.* 2011;6(8):1183-1191.
2. Liu L, et al. Cell type-differential modulation of prefrontal cortical GABAergic interneurons on low gamma rhythm and social interaction. *Sci Adv.* 2020;6(30):eaay4073.
3. Xu H, et al. A Disinhibitory Microcircuit Mediates Conditioned Social Fear in the Prefrontal Cortex. *Neuron.* 2019;102(3):668-682.
4. Wang J, et al. The basal forebrain to lateral habenula circuitry mediates social behavioral maladaptation. *Nat Commun.* 2024;15(1):4013.
5. Roth BL. DREADDs for Neuroscientists. *Neuron.* 2016;89(4):683-694.
6. Yizhar O, et al. Optogenetics in neural systems. *Neuron.* 2011;71(1):9-34.
7. Wang H, et al. A molecularly defined amygdala-independent tetra-synaptic forebrain-to-hindbrain pathway for odor-driven innate fear and anxiety. *Nat*

Neurosci. 2024;27(3):514-526.

8. Liu Y, et al. A circuit from dorsal hippocampal CA3 to parvafox nucleus mediates chronic social defeat stress-induced deficits in preference for social novelty. *Sci Adv.* 2022;8(8):eabe8828.

9. Bi Q, et al. Microglia-derived PDGFB promotes neuronal potassium currents to suppress basal sympathetic tonicity and limit hypertension. *Immunity.* 2022;55(8):1466-1482.

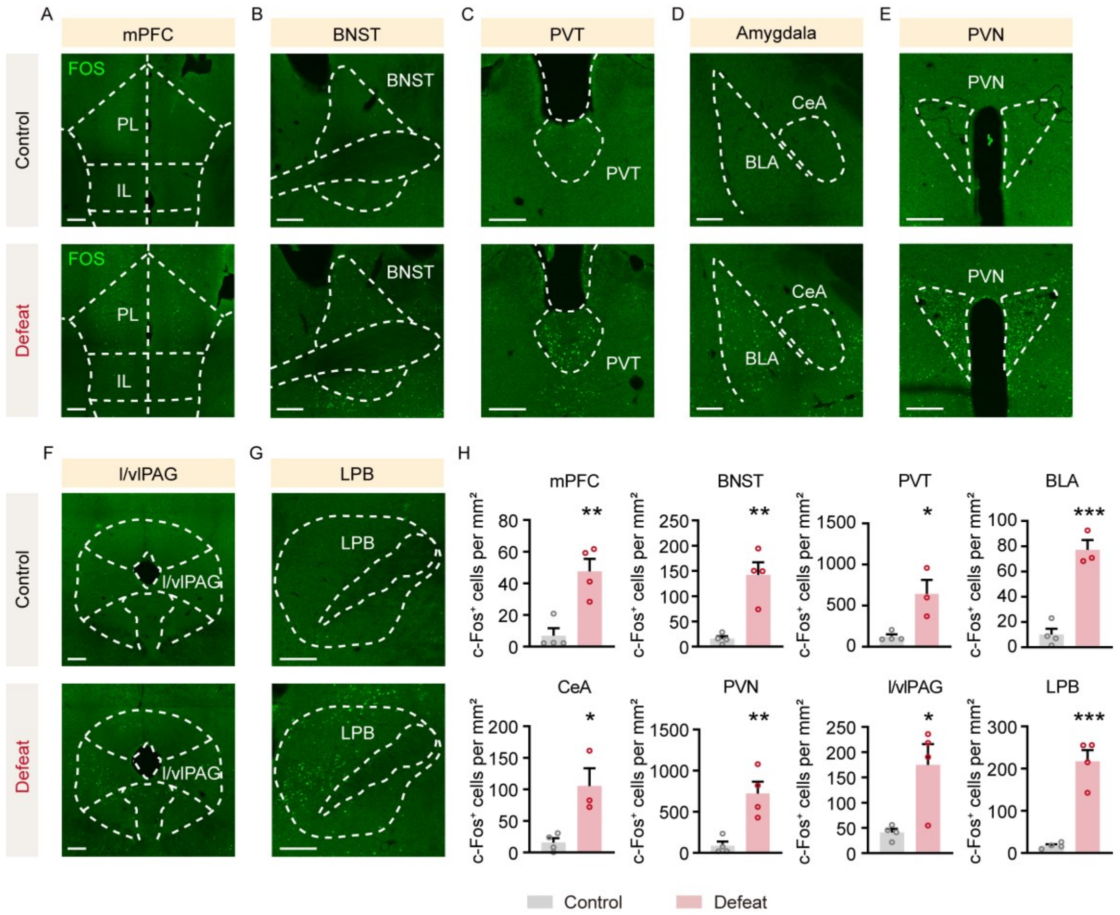

Supplementary Figure 1. Brain regions related to anxiety regulation are activated by acute social defeat stress. (**A-G**) Representative confocal images showing c-Fos positive cells from a control (top) and a social stress-exposed mouse (bottom). Scale bars, 200 µm. (**H**) Quantification of c-Fos positive cells in various brain regions. n = 4 mice for control group and n = 3 or 4 mice for social defeat group. Error bars indicate mean ± SEM. **P* < 0.05; ***P* < 0.01; ****P* < 0.001; two-tailed unpaired t test. mPFC, medial prefrontal cortex; PL, prelimbic cortex; IL, infralimbic cortex; BNST, bed nucleus of the stria terminalis; PVT, paraventricular thalamic nucleus; BLA, basolateral amygdala; CeA, central amygdala; PVN, paraventricular hypothalamic nucleus; l/vlPAG, lateral or ventrolateral periaqueductal gray; LPB, lateral parabrachial nucleus.

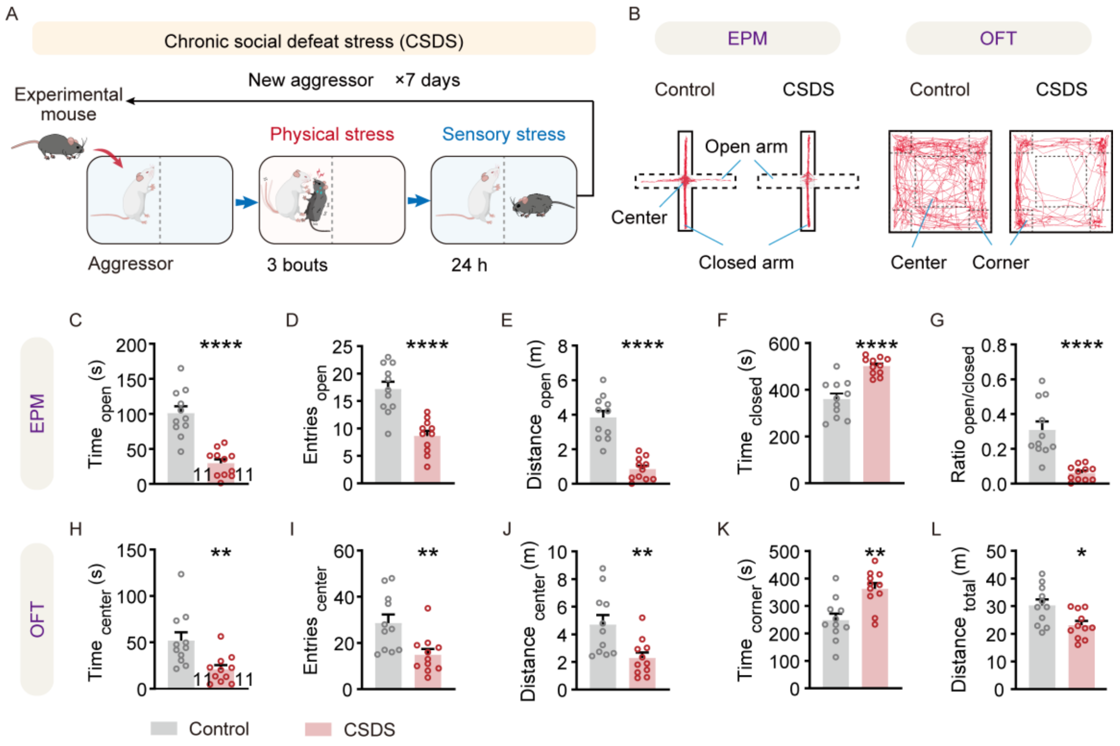

Supplementary Figure 2. CSDS induces robust anxiety-like behavior in mice. (**A**) Schematic illustration of the CSDS paradigm. (**B**) Representative movement traces of a control (left) and a CSDS mouse (right) in the EPM test or OFT. (**C-G**) Behavioral statistics of control and CSDS mice in the EPM test, including time spent (**C**), number of entries (**D**), distance traveled (**E**) in open arms, time spent in closed arms (**F**) and the open/closed ratio (time spent in open arms divided by time spent in closed arms) (**G**). (**H-L**) Behavioral statistics in the OFT, including time spent (**H**), number of entries (**I**), distance traveled (**J**) in the center zone, time spent in corner zones (**K**) and total distance traveled (**L**). n = 11 mice for each group. Error bars indicate mean ± SEM. *$P < 0.05$; **$P < 0.01$; ****$P < 0.0001$; two-tailed unpaired t test.

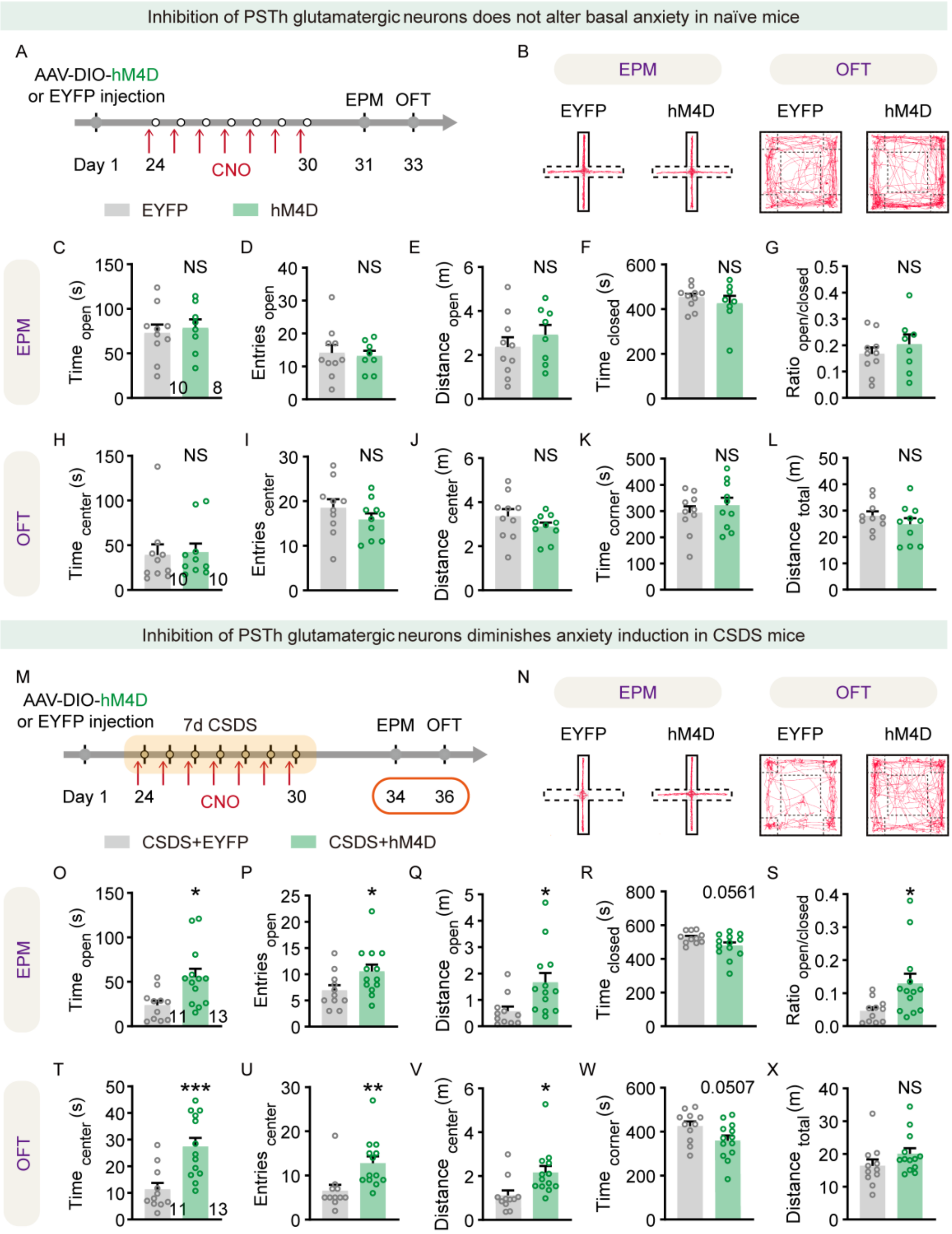

Supplementary Figure 3. Chronic inhibition of PSTh glutamatergic neurons does not alter basal anxiety-like behavior in naïve mice, but attenuates CSDS-induced anxiety-like behavior. (**A**) Experimental scheme illustrating chronic pharmacogenetic inhibition of PSTh^{Vglut2} neurons in naïve mice. (**B**) Representative movement traces in the EPM test or OFT. (**C-G**) Behavioral statistics of the EPM test, including time spent (**C**), number of entries (**D**), distance traveled (**E**) in open arms, time spent in closed arms (**F**) and the open/closed ratio (**G**). n = 10 mice for EYFP and n = 8 mice for hM4D. (**H-L**) Behavioral statistics of the OFT, including time spent (**H**), number of entries (**I**), distance traveled (**J**) in the center zone, time spent in corner zones (**K**) and total distance traveled (**L**). n = 10 mice for each group. (**M-X**) The same as (**A-L**)

but for chronic inhibition of $PSTh^{Vglut2}$ neurons during CSDS, with behavioral assessments conducted 3 days post-final CNO administration. n = 11 mice for CSDS+EYFP and n = 13 mice for CSDS+hM4D. Error bars indicate mean ± SEM. NS indicates no significant difference. *$P < 0.05$; **$P < 0.01$; ***$P < 0.001$; two-tailed unpaired t test.

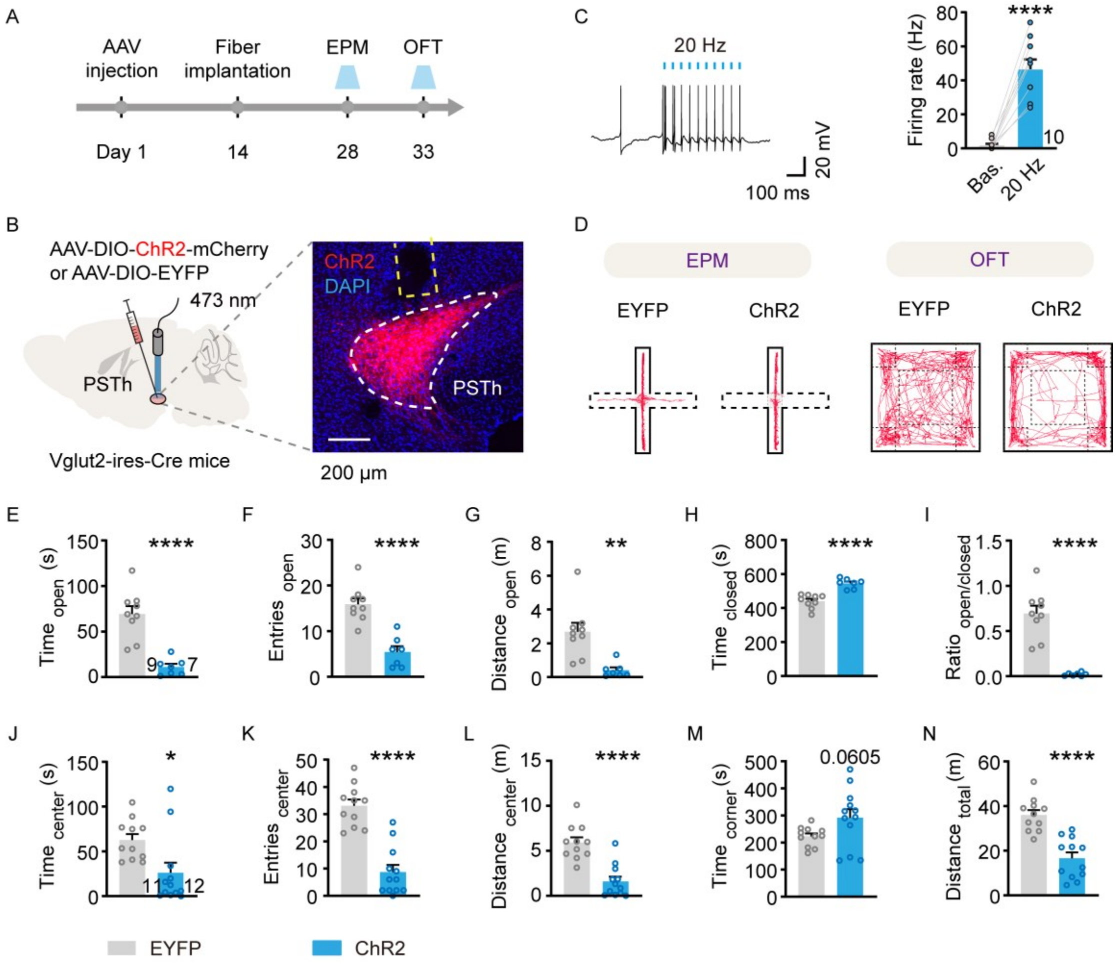

Supplementary Figure 4. Optogenetic activation of PSTh glutamatergic neurons induces anxiety-like behavior in naïve mice. (**A**) Experimental illustration showing optogenetic activation of PSTh^{Vglut2} neurons during anxiety-like behavioral assays in naïve mice. (**B**) Schematic description and representative image of ChR2 expression. Scale bar, 200 µm. (**C**) Representative traces (left) and statistical comparison of spike firing (right) before and after light stimulations. n = 10 neurons from 2 mice. (**D**) Representative movement traces in the EPM test or OFT. (**E-I**) Behavioral statistics of the EPM test, including time spent (**E**), number of entries (**F**), distance traveled (**G**) in open arms, time spent in closed arms (**H**) and the open/closed ratio (**I**). n = 9 mice for EYFP and n = 7 mice for ChR2. (**J-N**) Behavioral statistics of the OFT, including time spent (**J**), number of entries (**K**), distance traveled (**L**) in the center zone, time spent in corner zones (**M**) and total distance traveled (**N**). n = 11 mice for EYFP and n = 12 mice for ChR2. Error bars indicate mean ± SEM. *$P < 0.05$; **$P < 0.01$; ****$P < 0.0001$; two-tailed unpaired t test.

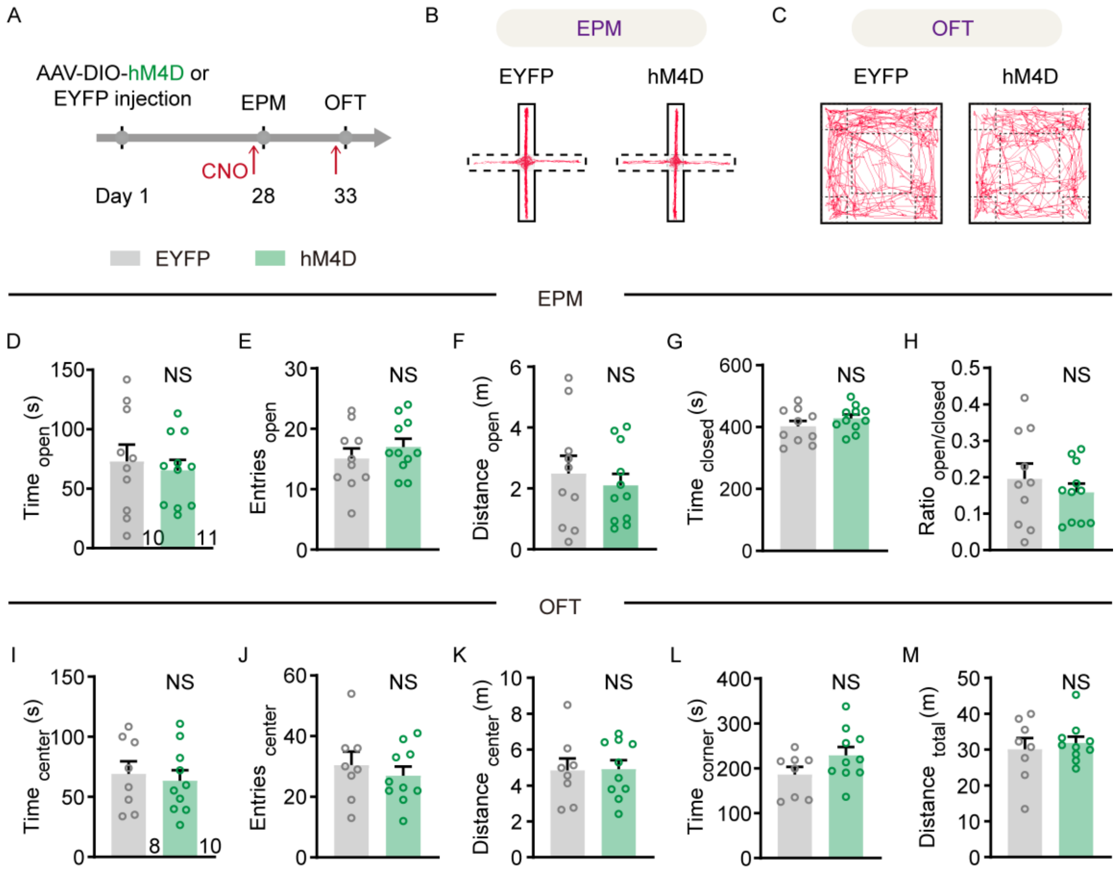

Supplementary Figure 5. Pharmacogenetic inhibition of PSTh glutamatergic neurons has no effect on basal anxiety-like behavior of non-stressed mice. (**A**) Experimental scheme illustrating the effect of pharmacogenetic inhibition of $PSTh^{Vglut2}$ neurons on basal anxiety-like behavior in naïve mice. (**B** and **C**) Representative movement traces showing locations of an EYFP (left) and a hM4D mouse (right) in the EPM test (**B**) or OFT (**C**). (**D-H**) Behavioral statistics of EYFP and hM4D mice in the EPM test, including time spent (**D**), number of entries (**E**), distance traveled (**F**) in open arms, time spent in closed arms (**G**) and the open/closed ratio (**H**). n = 10 mice for EYFP and n =11 mice for hM4D. (**I-M**) Behavioral statistics of EYFP and hM4D mice in the OFT, including time spent (**I**), number of entries (**J**), distance traveled (**K**) in the center zone, time spent in corner zones (**L**) and total distance traveled (**M**). n = 8 mice for EYFP and n =10 mice for hM4D. Error bars indicate mean ± SEM. NS indicates no significant difference by two-tailed unpaired t test.

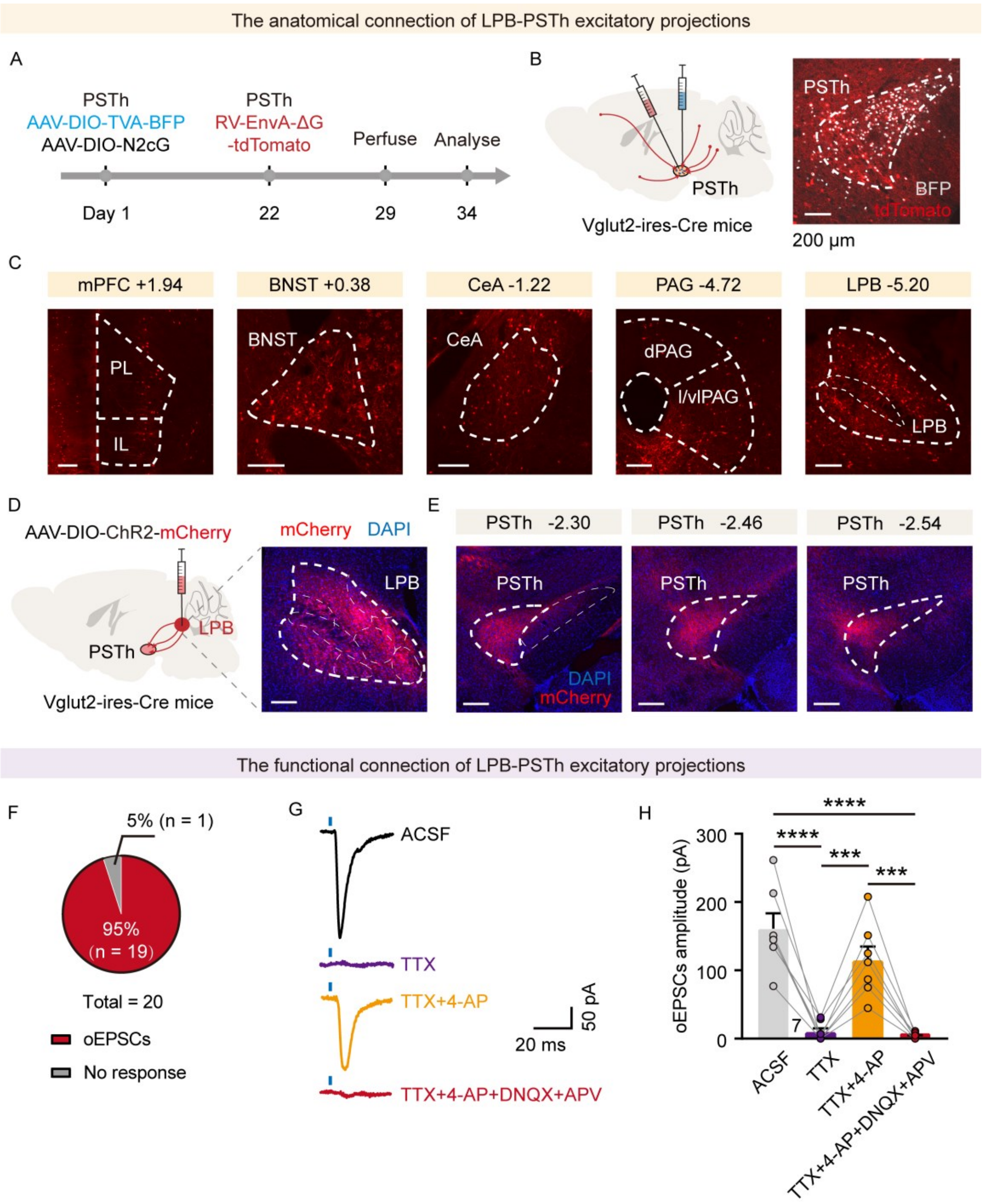

Supplementary Figure 6. The anatomical and functional connections of excitatory LPB-PSTh pathways. (**A** and **B**) Flow diagram (**A**) and schematic diagram (**B**, left) illustrating the pseudotyped rabies injection for tracing monosynaptic inputs to PSTh^{Vglut2} neurons. (**B**, right) RV expression in the PSTh. Scale bar, 200 μm. (**C**) Representative images of RV retrogradely labeled neurons. Scale bars, 200 μm. (**D**) Schematic diagram and representative image of ChR2-mCherry expression in LPB^{Vglut2} neurons. Scale bar, 200 μm. (**E**) Example images of mCherry-targeted axon terminals in PSTh. Scale bars, 200 μm. (**F**) The number and proportion of recorded PSTh^{Vglut2} neurons that exhibited light-evoked EPSCs. n = 20 neurons from 2 mice. (**G** and **H**) Representative traces (**G**) and quantifications (**H**) of light-evoked EPSCs recorded from PSTh^{Vglut2} neurons under bath of ACSF (black line), and with the addition of TTX (purple line), TTX + 4-AP (orange line), or TTX + 4-AP + DNQX + APV (red line). Blue lines indicate light pulse stimulation

(1 ms). n = 7 neurons from 2 mice. Error bars indicate mean ± SEM. ***$P < 0.001$; ****$P < 0.0001$; one-way ANOVA, Tukey multiple comparison post hoc tests.

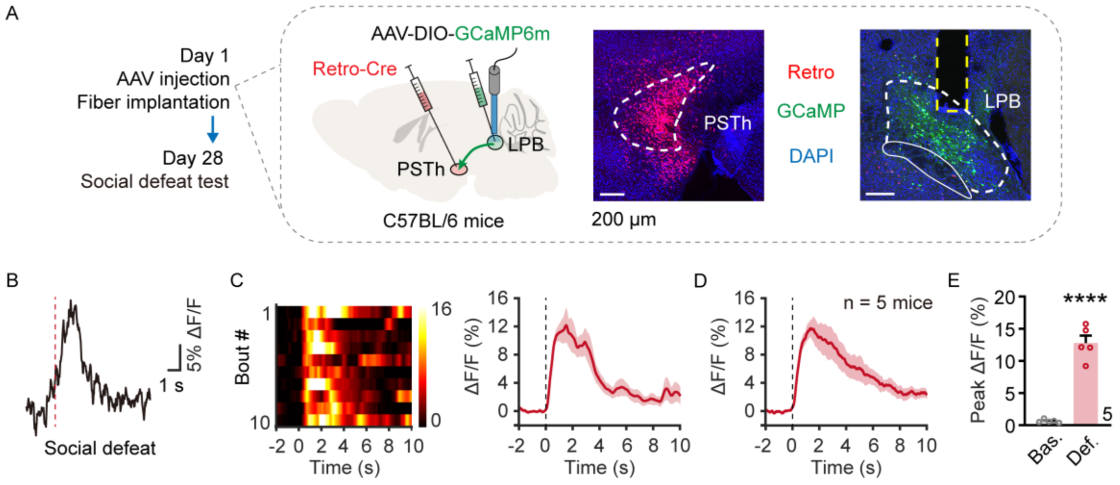

Supplementary Figure 7. Social defeat stress strongly activates the LPB-PSTh pathway. (**A**) Left: A flow diagram and schematic diagram illustrating fiber photometry recording of the LPB-PSTh pathway. Right: Example microscopy images showing Retro-Cre expression in the PSTh, as well as GCaMP6m expression and optical fiber placement in LPB. Scale bars, 200 μm. (**B**) Representative raw trace showing GCaMP6m fluorescence changes evoked by social defeat. (**C**) Left: Heatmaps illustrating LPB-PSTh circuitry calcium signals aligned to each bout of defeat stress (each row) from an example mouse. Right: The peri-event plot of average calcium transients from a defeated mouse. The thick line indicates mean and the shaded area indicates SEM. The dotted line marks the onset of social defeat. (**D**) The peri-event plot of the mean calcium transients under social defeat for entire test group (n=5). (**E**) Statistical comparison of peak fluorescence signals before and after social defeat (n = 5). Error bars indicate mean ± SEM. ****$P < 0.0001$; two-tailed unpaired t test.

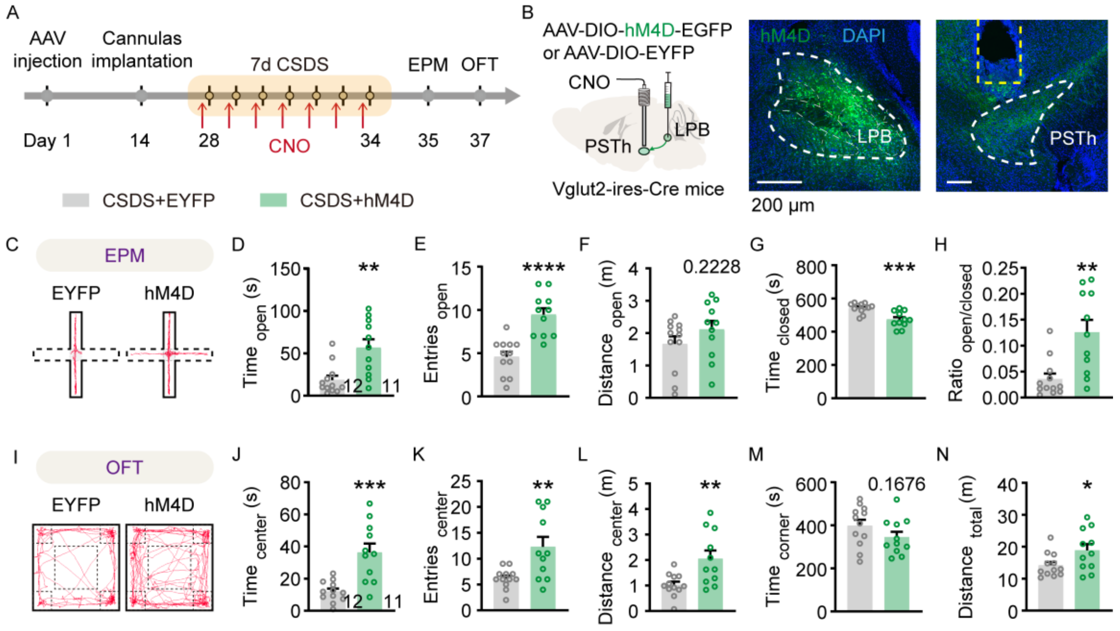

Supplementary Figure 8. Chronic inhibition of the LPB-PSTh pathway alleviates CSDS-induced anxiety-like behavior. (**A**) Experimental scheme illustrating the effect of chronic pharmacogenetic inhibition of LPB-PSTh excitatory projections on CSDS-induced anxiety-like behavior. (**B**) Schematic description and representative images of hM4D expression in LPB glutamatergic neurons, as well as their axon terminals and guide cannula implantation in PSTh. Scale bars, 200 μm. (**C** and **I**) Representative movement traces of an EYFP (left) and a hM4D mouse (right) in the EPM test (**C**) or OFT (**I**) post-CSDS. (**D-H**) Behavioral statistics of the EPM test, including time spent (**D**), number of entries (**E**), distance traveled (**F**) in open arms, time spent in closed arms (**G**) and the open/closed ratio (**H**). (**J-N**) Behavioral statistics of the OFT, including time spent (**J**), number of entries (**K**), distance traveled (**L**) in the center zone, time spent in corner zones (**M**) and total distance traveled (**N**). n = 12 mice for CSDS+EYFP and n = 11 mice for CSDS+hM4D group. Error bars indicate mean ± SEM. **P* < 0.05; ***P* < 0.01; ****P* < 0.001; *****P* < 0.0001; two-tailed unpaired t test.

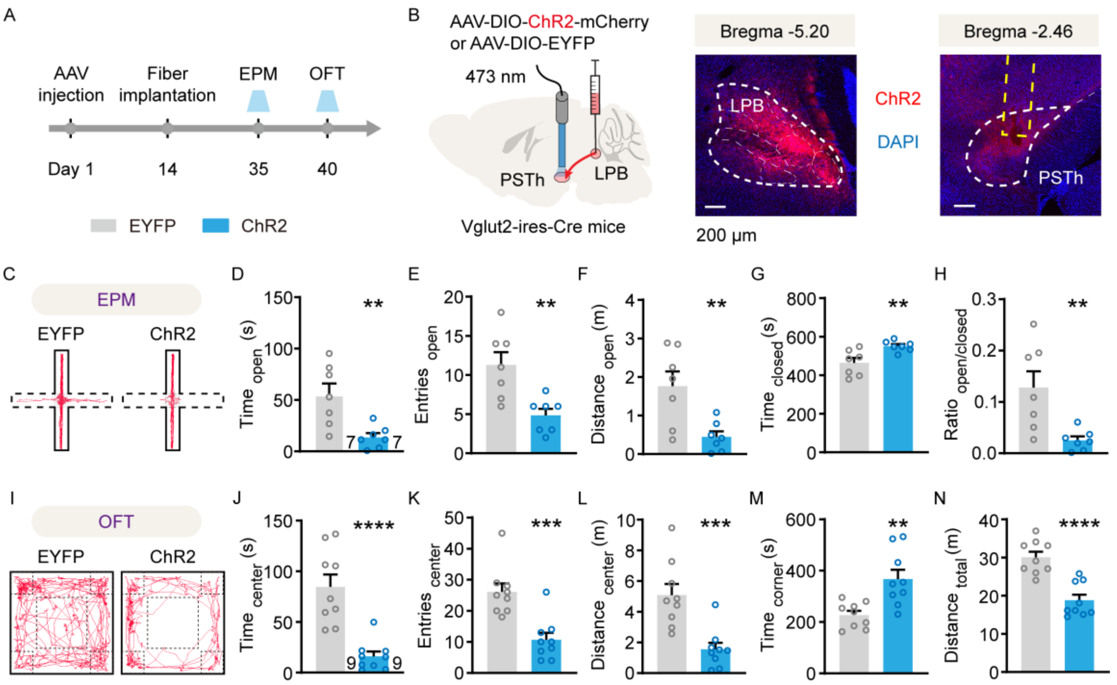

Supplementary Figure 9. Optogenetic activation of LPB-PSTh excitatory projections promotes anxiety-like behavior in naïve mice. (**A**) Experimental illustration showing optogenetic activation of the LPB-PSTh pathway during anxiety-like behavioral assays in naïve mice. (**B**) Schematic description and representative images of ChR2 expression in LPB glutamatergic neurons, as well as their axon terminals and optical fiber implantation in PSTh. Scale bars, 200 μm. (**C** and **I**) Representative movement traces of an EYFP (left) and a ChR2 mouse (right) in the EPM test (**C**) or OFT (**I**). (**D-H**) Behavioral statistics of the EPM test, including time spent (**D**), number of entries (**E**), distance traveled (**F**) in open arms, time spent in closed arms (**G**) and the open/closed ratio (**H**). n = 7 mice for each group. (**J-N**) Behavioral statistics of the OFT, including time spent (**J**), number of entries (**K**), distance traveled (**L**) in the center zone, time spent in corner zones (**M**) and total distance traveled (**N**). n = 9 mice for each group. Error bars indicate mean ± SEM. $**P < 0.01$; $***P < 0.001$; $****P < 0.0001$; two-tailed unpaired t test.

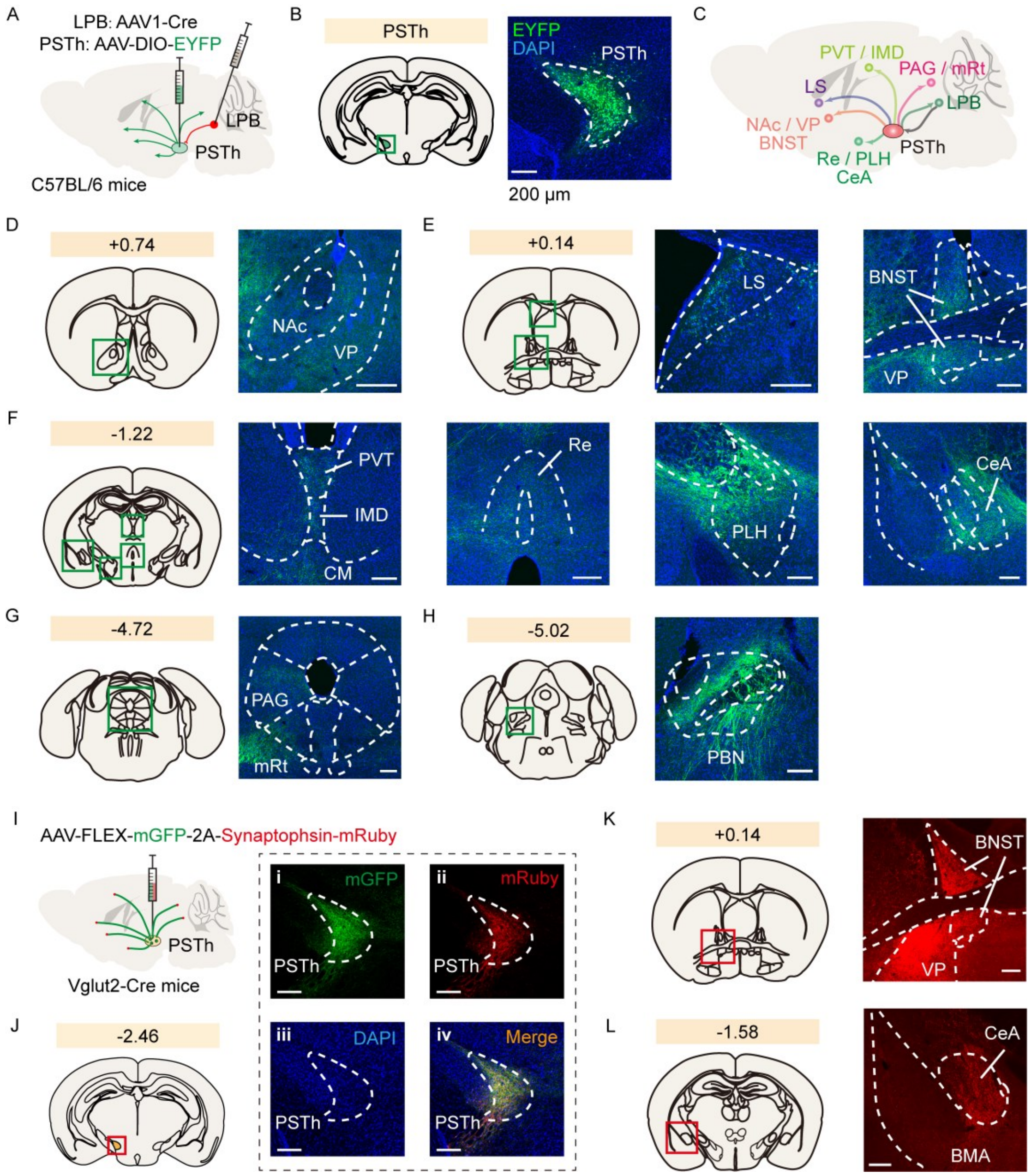

Supplementary Figure 10. Mapping targets of the LPB-PSTh circuit. (**A**) A schematic diagram of the anterograde virus injection (AAV1-Cre) in the LPB and AAV-DIO-EFYP infusion in the PSTh for tracing outputs of the LPB-PSTh pathway. (**B**) Representative image showing EYFP-labeled PSTh neurons that are innervated by LPB. Scale bar, 200 μm. (**C**) A summarized diagram showing output targets of the LPB-PSTh pathway. (**D-H**) Example images of EYFP-labeled axons in downstream structures. Scale bars, 200 μm. (**I**) A schematic diagram of virus injection (AAV-FLEX-mGFP-2A-Synaptophysin-mRuby) for tracing downstream structures of $PSTh^{Vglut2}$ neurons. (**J**) Representative images showing anterograde virus-labeled PSTh glutamatergic neurons (i-iv). Scale bars, 200 μm. (**K** and **L**) Representative images showing anterograde terminals of PSTh glutamatergic neurons in the BNST (**K**) and CeA (**L**). Scale bars, 200 μm. NAc, nucleus accumbens; VP, ventral pallidum; LS, lateral septal nucleus; IMD, intermediodorsal thalamic nucleus; CM, central medial thalamic nucleus; Re, reuniens thalamic nucleus; PLH, peduncular part of the lateral hypothalamus; mRt, mesencephalic reticular formation; BMA, basomedial amygdaloid nucleus.

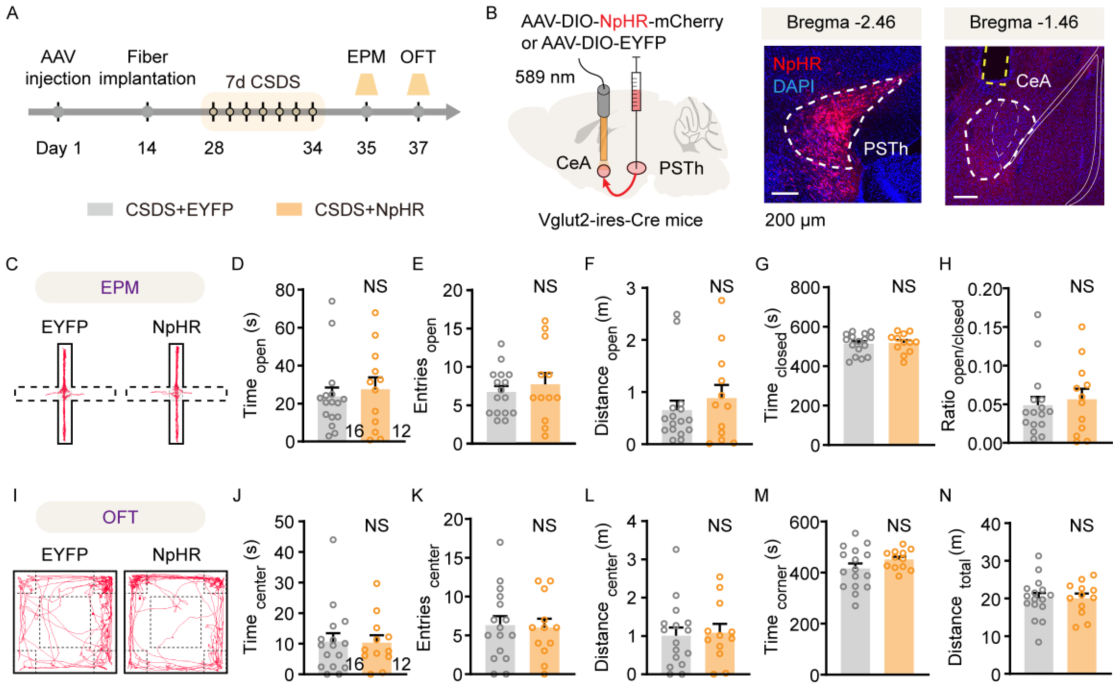

Supplementary Figure 11. Behavioral results of optogenetic inhibition of the PSTh-CeA excitatory pathway. (**A**) Experimental scheme showing optogenetic suppression of PSTh-CeA excitatory projections during anxiety-like behavioral tests in CSDS mice. (**B**) Schematic description and representative images of NpHR expression in PSTh glutamatergic neurons, as well as their axon terminals and optical fiber in the CeA. Scale bars, 200 µm. (**C** and **I**) Representative movement traces of an EYFP mouse (left) and a NpHR (right) in the EPM test (**C**) or OFT (**I**) post-CSDS. (**D-H**) Behavioral statistics of the EPM test, including time spent (**D**), number of entries (**E**), distance traveled (**F**) in open arms, time spent in closed arms (**G**) and the open/closed ratio (**H**). (**J-N**) Behavioral statistics of the OFT, including time spent (**J**), number of entries (**K**), distance traveled (**L**) in the center zone, time spent in corner zones (**M**) and total distance traveled (**N**). n = 16 mice for CSDS+EYFP and n = 12 for CSDS+NpHR group. Error bars indicate mean ± SEM. NS indicates no significant difference by two-tailed unpaired t test.

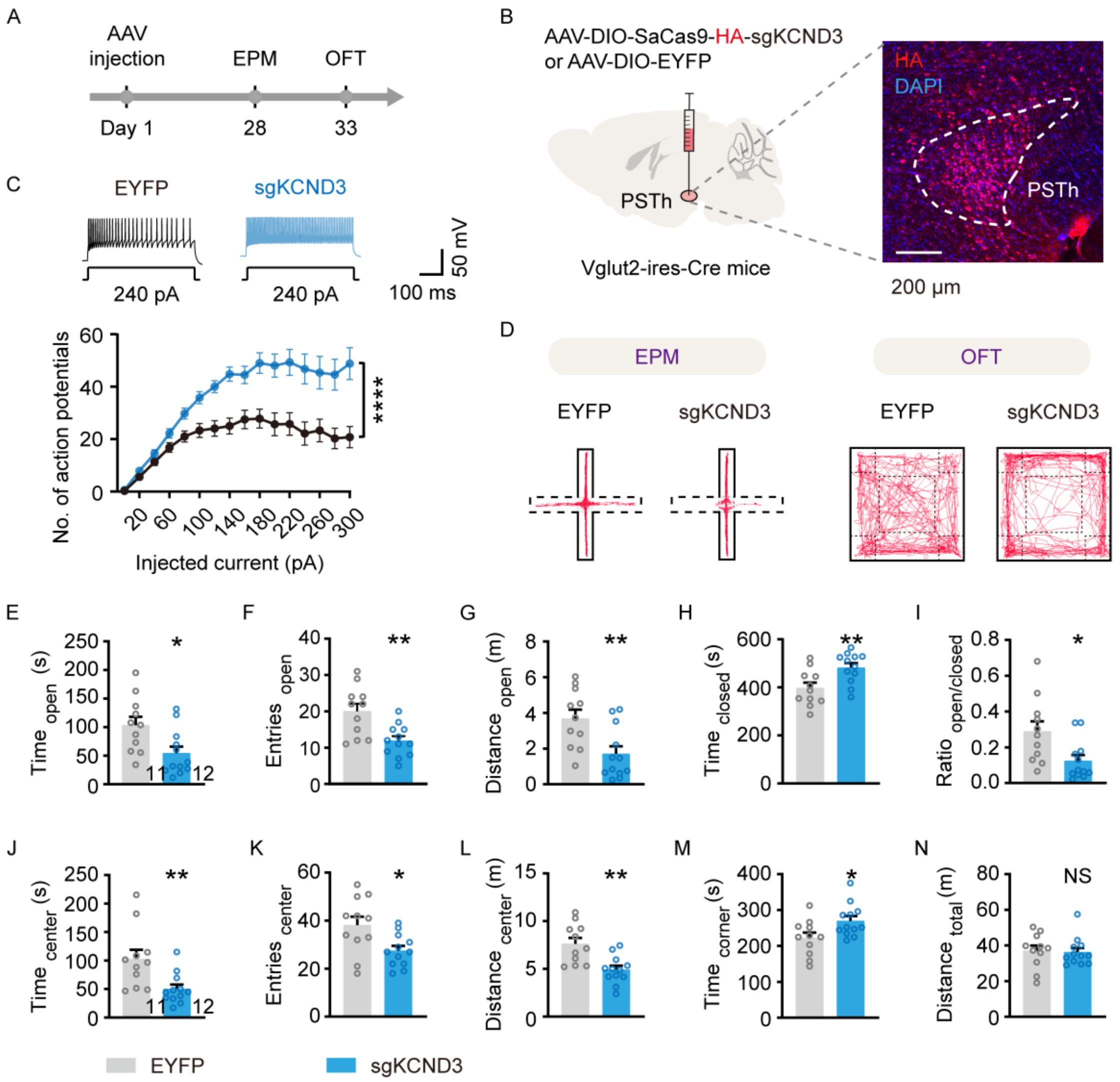

Supplementary Figure 12. Kv4.3 downregulation in PSTh glutamatergic neurons elicits anxiety-like behavior. (**A**) Experimental flow chart. (**B**) Schematic description and representative image of SaCas9-sgKCND3 expression. Scale bar, 200 μm. (**C**) Representative traces (top) and statistical comparison of spike firing (bottom) of EYFP (dark) and sgKCND3-expressed (blue) PSTh^{Vglut2} neurons. n = 12 neurons from 4 mice for EYFP, n = 13 neurons from 3 mice for sgKCND3. (**D**) Representative movement traces in the EPM test or OFT. (**E-I**) Behavioral statistics of the EPM test, including time spent (**E**), number of entries (**F**), distance traveled (**G**) in open arms, time spent in closed arms (**H**) and the open/closed ratio (**I**). (**J-N**) Behavioral statistics of the OFT, including time spent (**J**), number of entries (**K**), distance traveled (**L**) in the center zone, time spent in corner zones (**M**) and total distance traveled (**N**). n = 11 mice for EYFP and n = 12 mice for sgKCND3 group. Error bars indicate mean ± SEM. NS indicates no significant difference. *$P < 0.05$; **$P < 0.01$; ****$P < 0.0001$; two-way ANOVA, Bonferroni multiple comparison post hoc tests in **C**; two-tailed unpaired t test in **E-N**.
